# Supplementary material for: The Antitumor Effect of Caffeic Acid Phenethyl Ester by Downregulating Mucosa-Associated Lymphoid Tissue 1 via AR/p53/NF-κB Signaling in Prostate Carcinoma Cells
Source: Cancers (Basel). 2022 Jan 6;14(2):274. doi: 10.3390/cancers14020274 (PMC8773797; doi:10.3390/cancers14020274)
Supplement: Supplementary file 1 [file cancers-14-00274-s001.zip › cancers-1524042-supplementary/Figure S1.pdf]

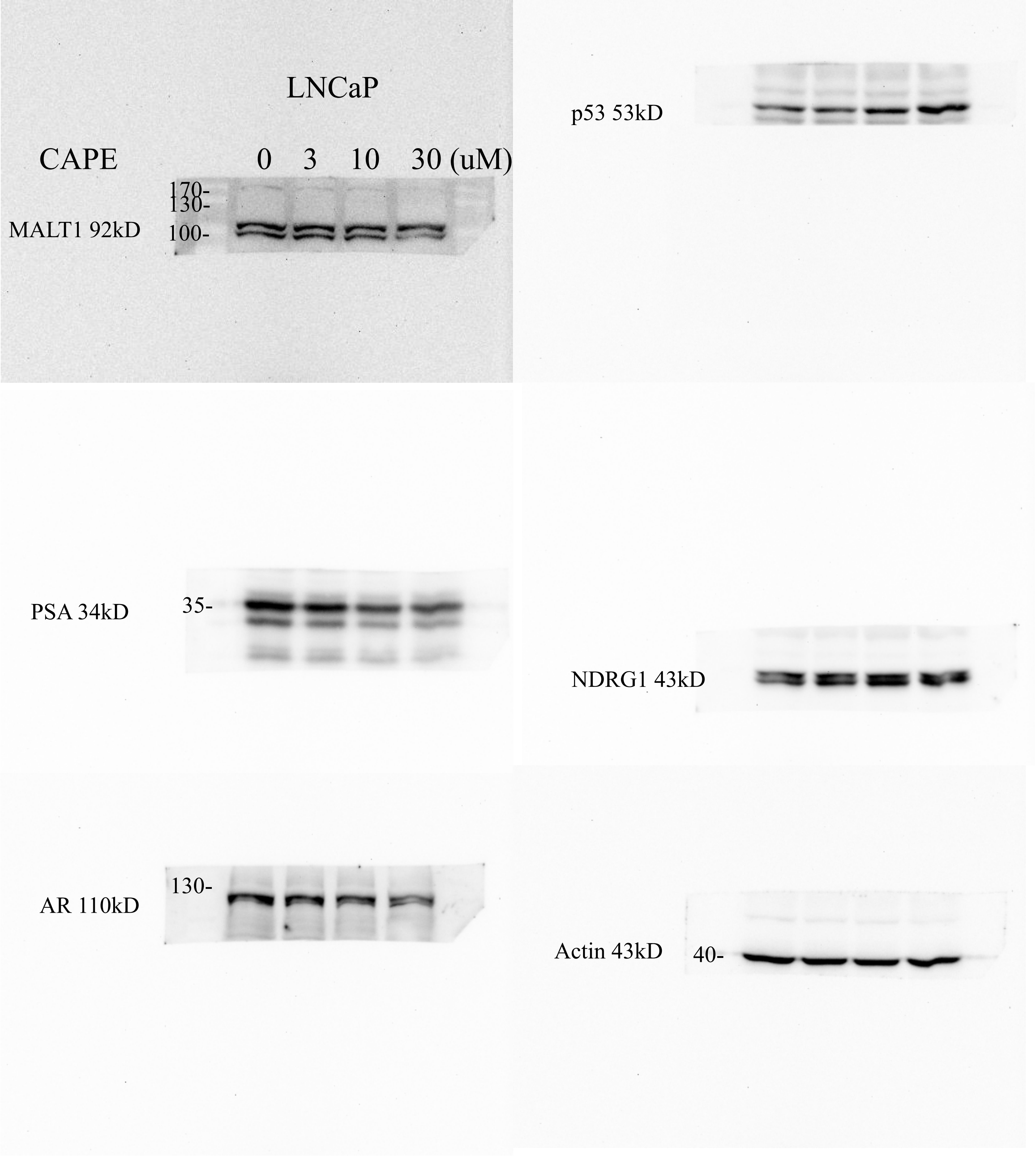
LNCaP
CAPE
0 3 10 30 (uM)
170-
130-
MALT1 92kD
100-
p53 53kD
PSA 34kD
35-
NDRG1 43kD
AR 110kD
130-
Actin 43kD
40-

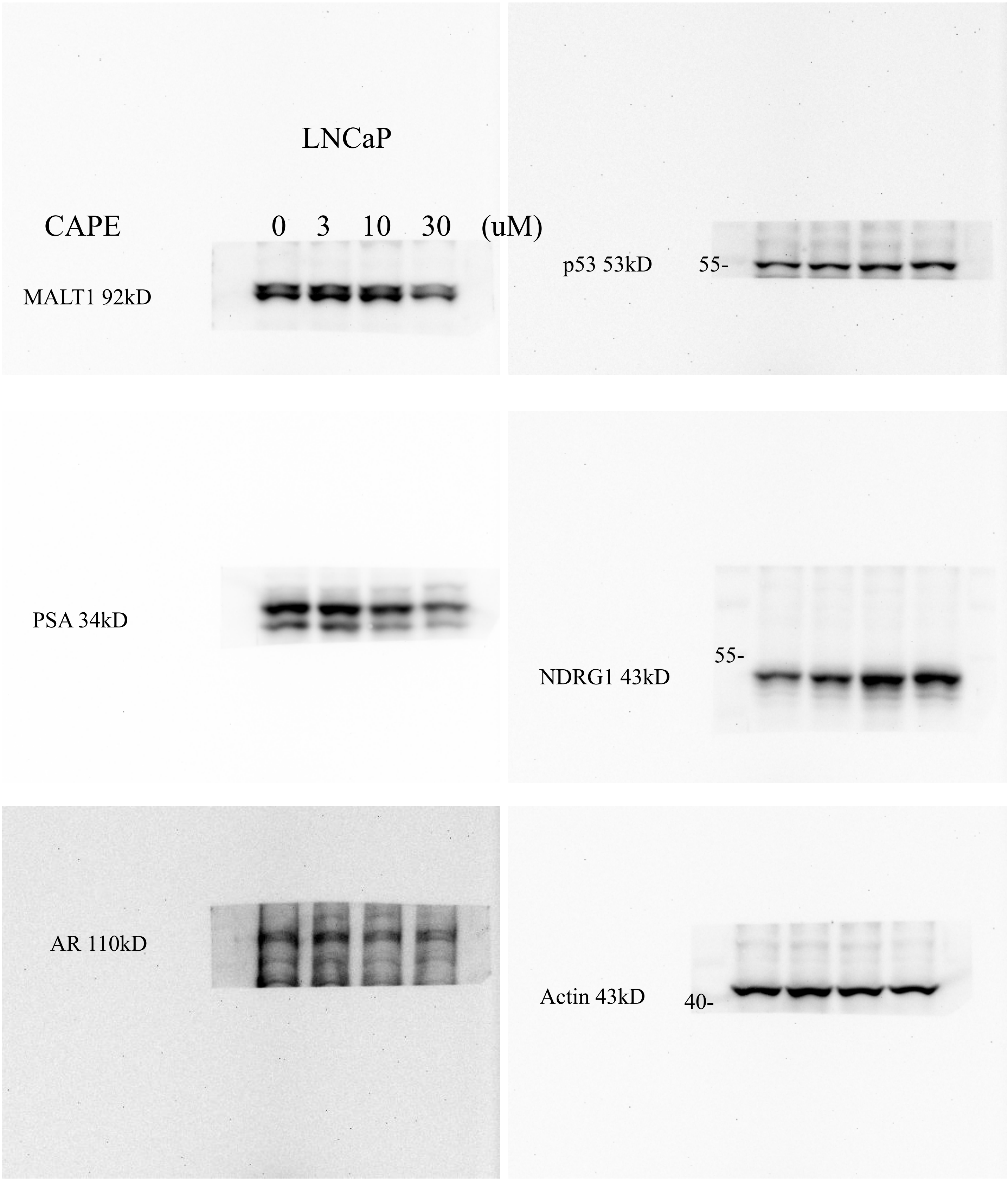
LNCaP
CAPE
0 3 10 30 (uM)
MALT1 92kD
p53 53kD
55-
PSA 34kD
55-
NDRG1 43kD
AR 110kD
Actin 43kD
40-

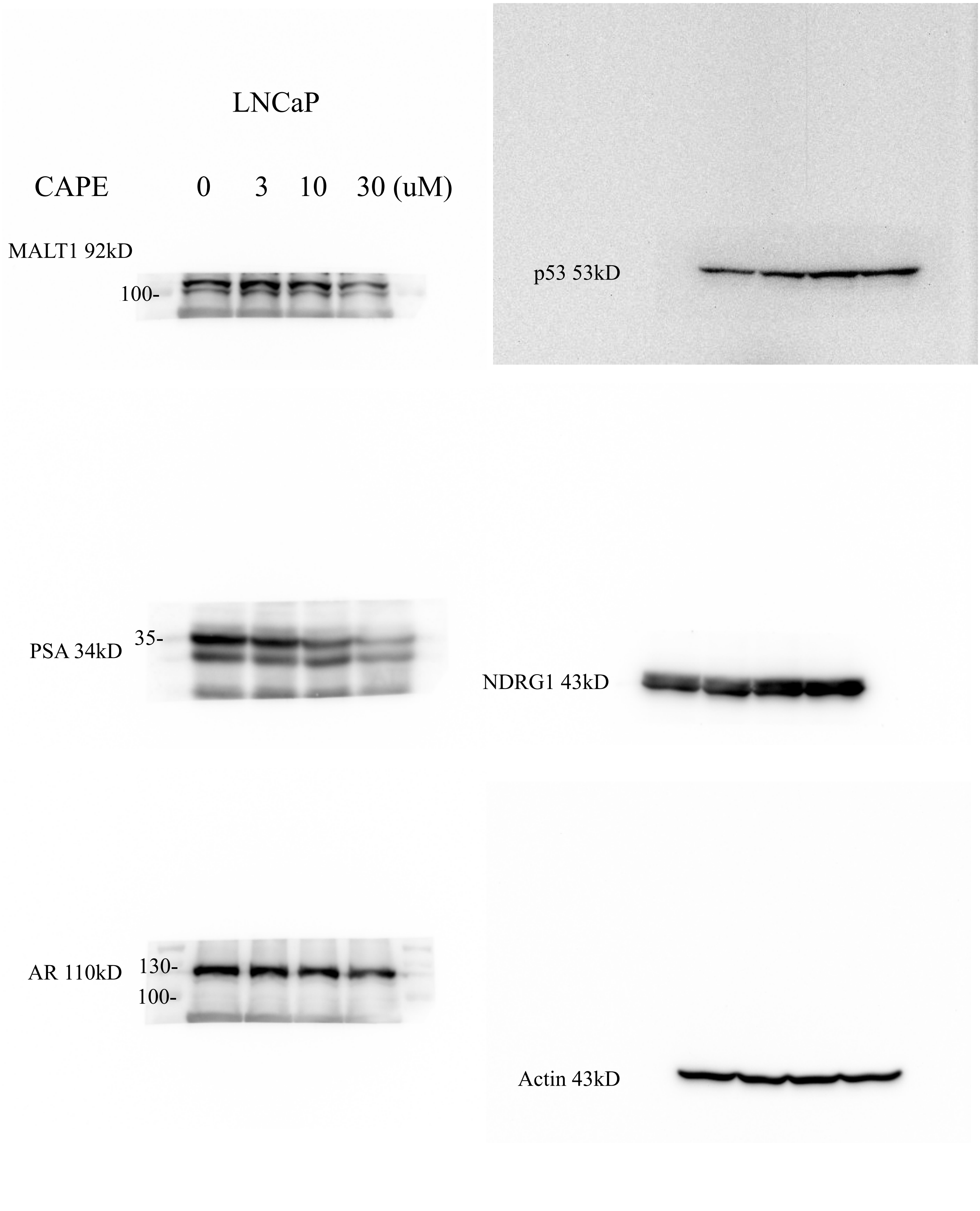
LNCaP
CAPE
0
3
10
30 (uM)
MALT1 92kD
100-
p53 53kD
PSA 34kD
35-
NDRG1 43kD
AR 110kD
130-
100-
Actin 43kD

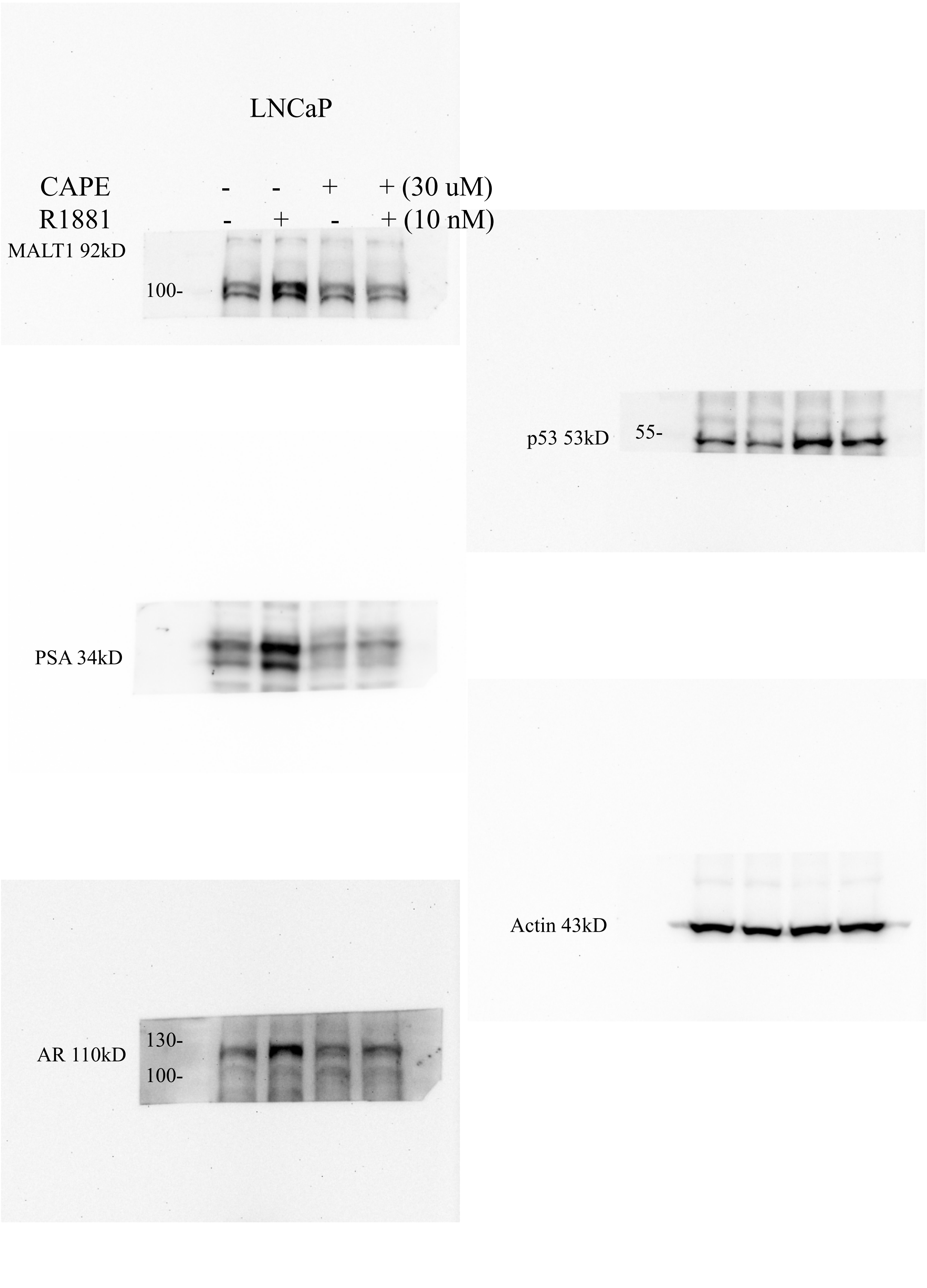
LNCaP
CAPE - - + + (30 uM)
R1881 - + - + (10 nM)
MALT1 92kD
100-
p53 53kD
55-
PSA 34kD
Actin 43kD
AR 110kD
130-
100-

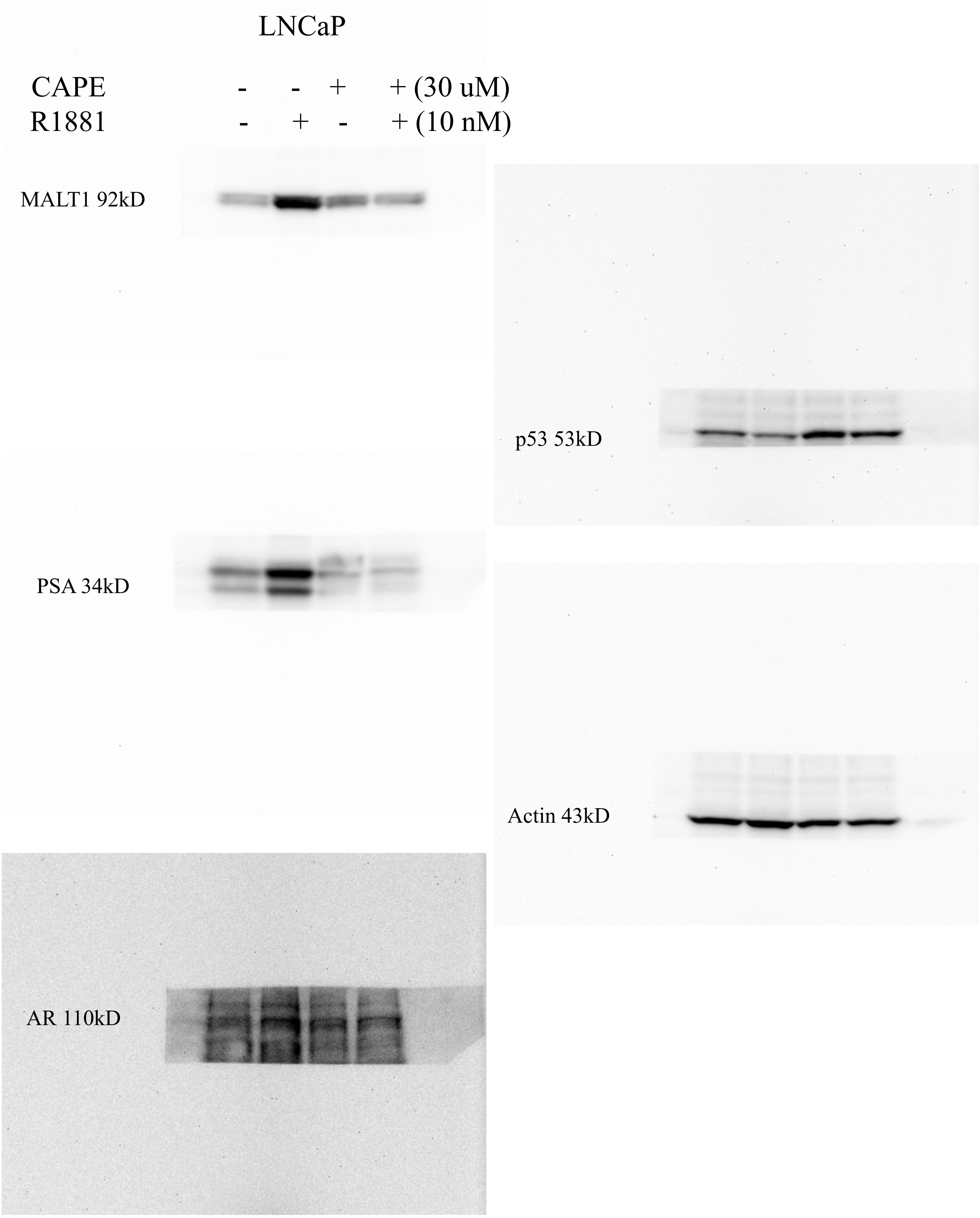
LNCaP
CAPE - - + + (30 uM)
R1881 - + - + (10 nM)
MALT1 92kD
PSA 34kD
AR 110kD
p53 53kD
Actin 43kD

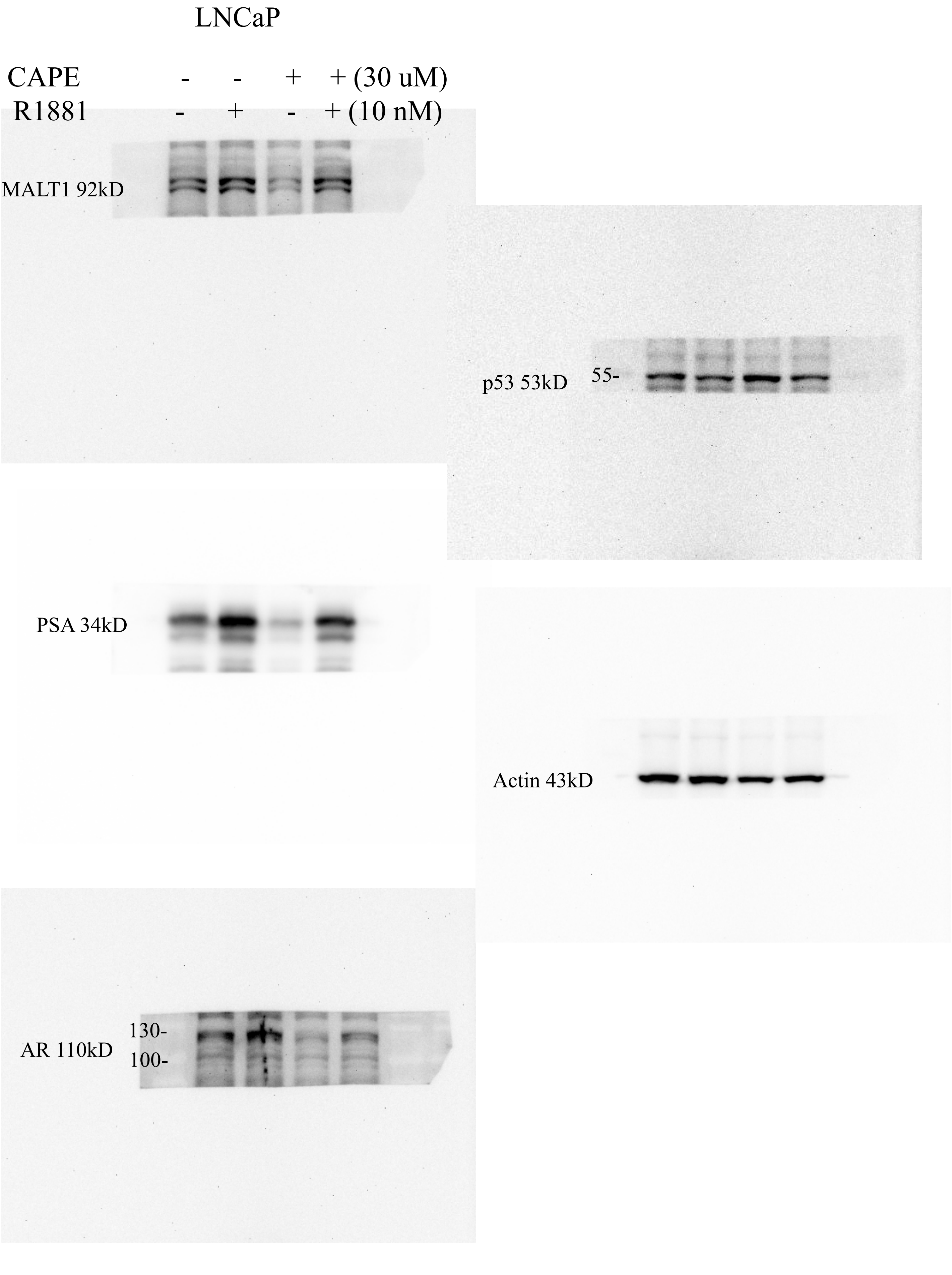
LNCaP
CAPE - - + + (30 uM)
R1881 - + - + (10 nM)
MALT1 92kD
p53 53kD
55-
PSA 34kD
Actin 43kD
AR 110kD
130-
100-

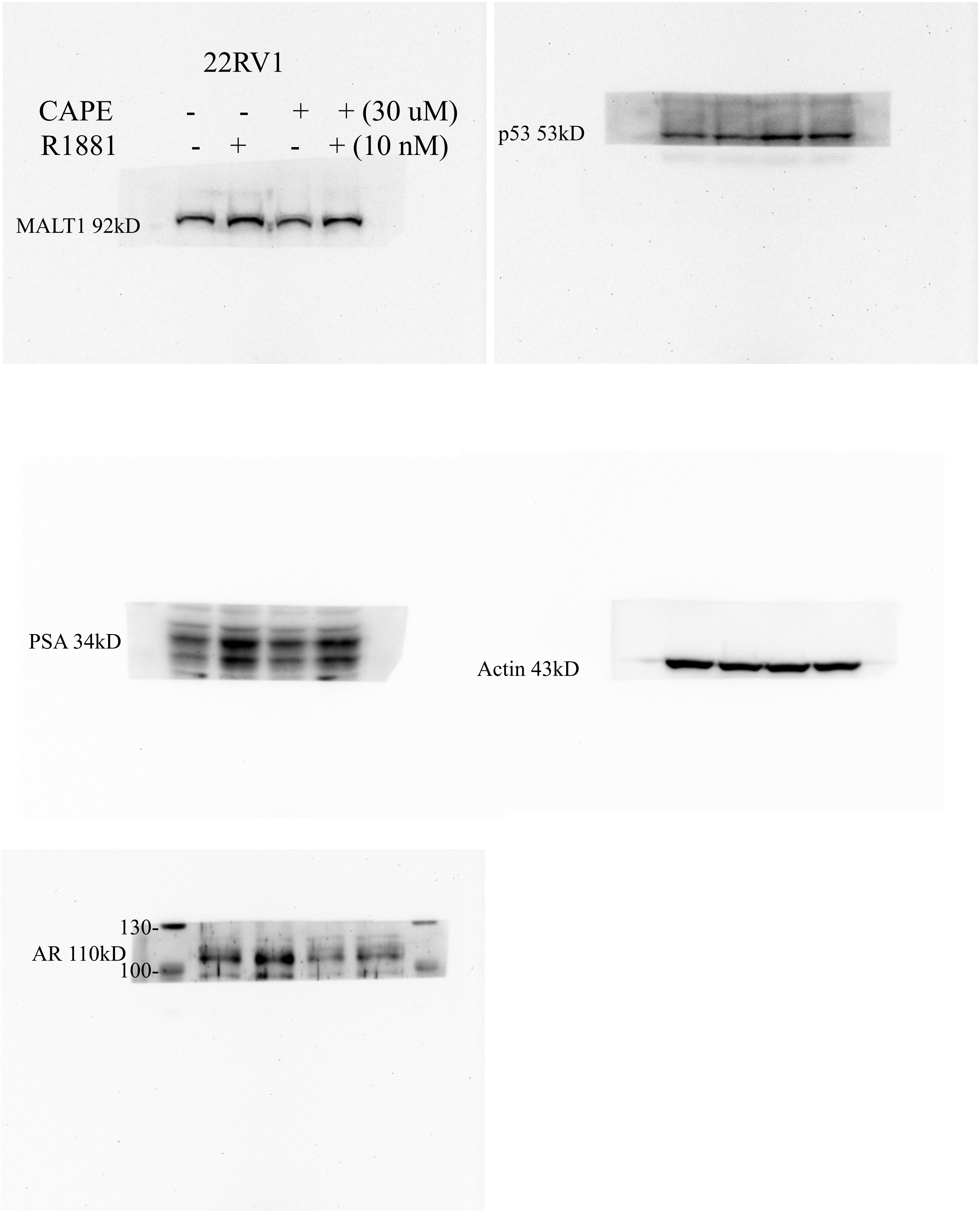
22RV1
CAPE - - + + (30 uM)
R1881 - + - + (10 nM)
MALT1 92kD
p53 53kD
PSA 34kD
Actin 43kD
130-
AR 110kD
100-

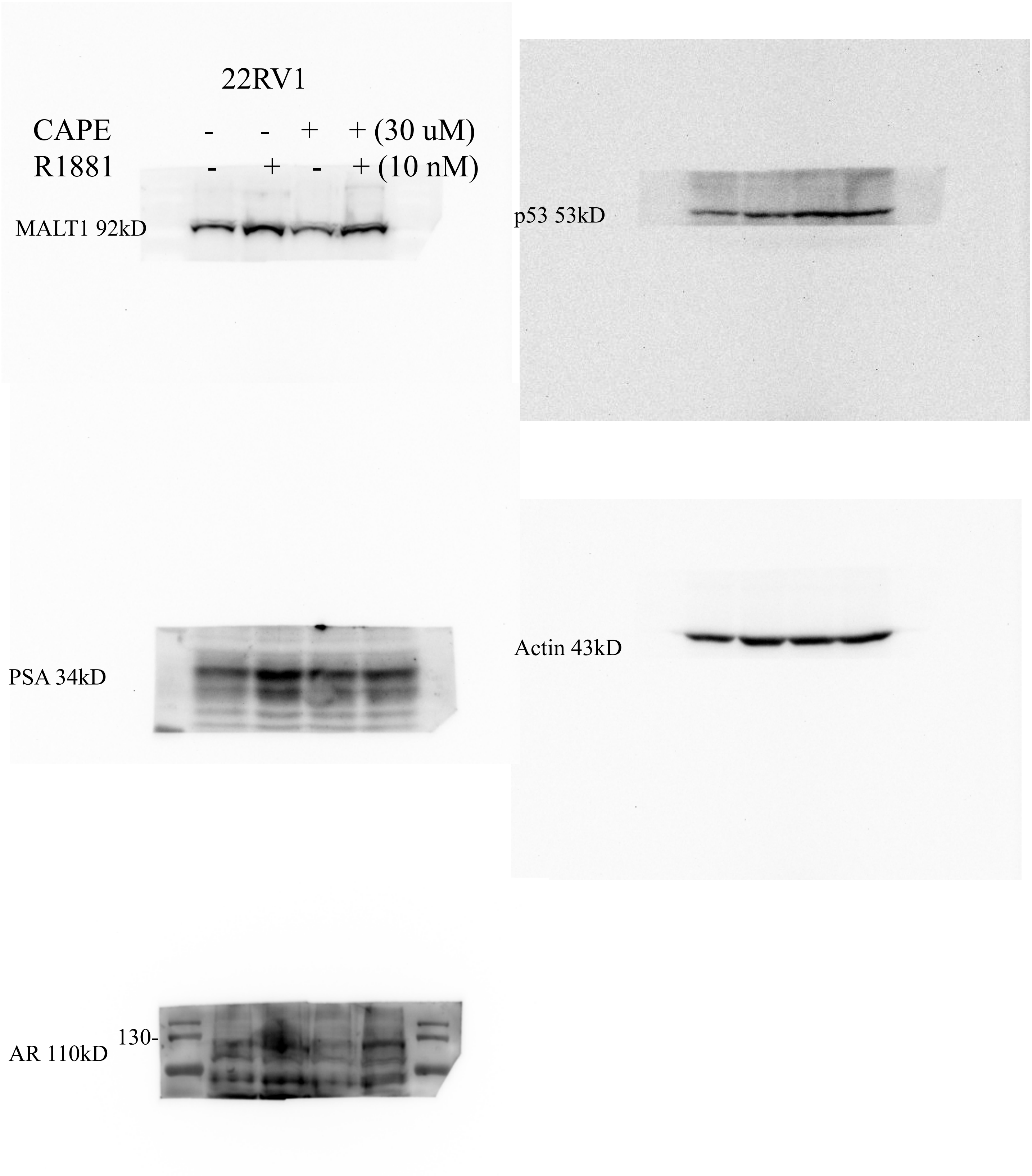
22RV1
CAPE - - + + (30 uM)
R1881 - + - + (10 nM)
MALT1 92kD
p53 53kD
PSA 34kD
Actin 43kD
AR 110kD
130-

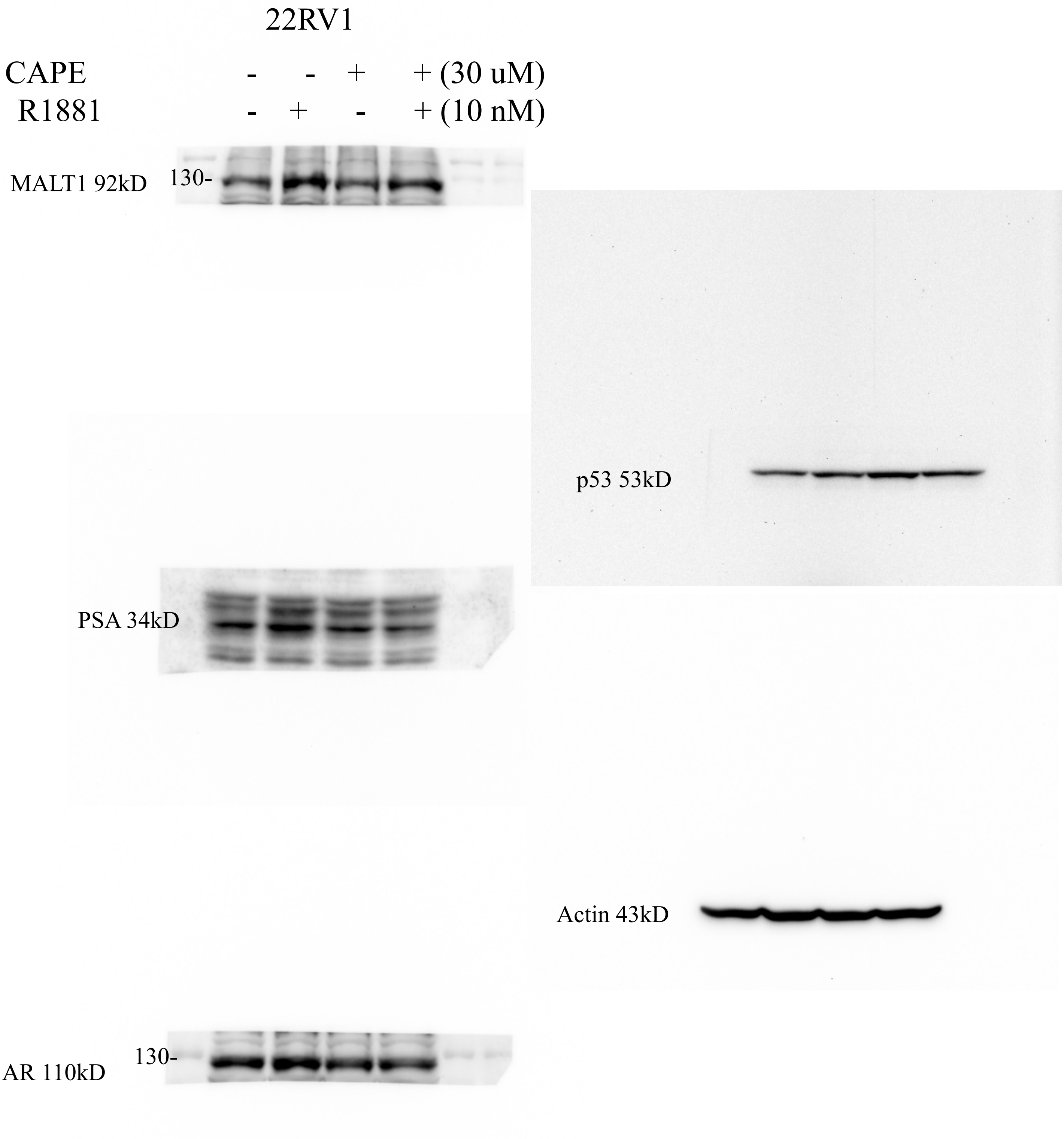

Figure S1: Original uncropped Western blots of figure 1
