# Supplementary material for: The Antitumor Effect of Caffeic Acid Phenethyl Ester by Downregulating Mucosa-Associated Lymphoid Tissue 1 via AR/p53/NF-κB Signaling in Prostate Carcinoma Cells
Source: Cancers (Basel). 2022 Jan 6;14(2):274. doi: 10.3390/cancers14020274 (PMC8773797; doi:10.3390/cancers14020274)
Supplement: Supplementary file 1 [file cancers-14-00274-s001.zip › cancers-1524042-supplementary/Figure S3.pdf]

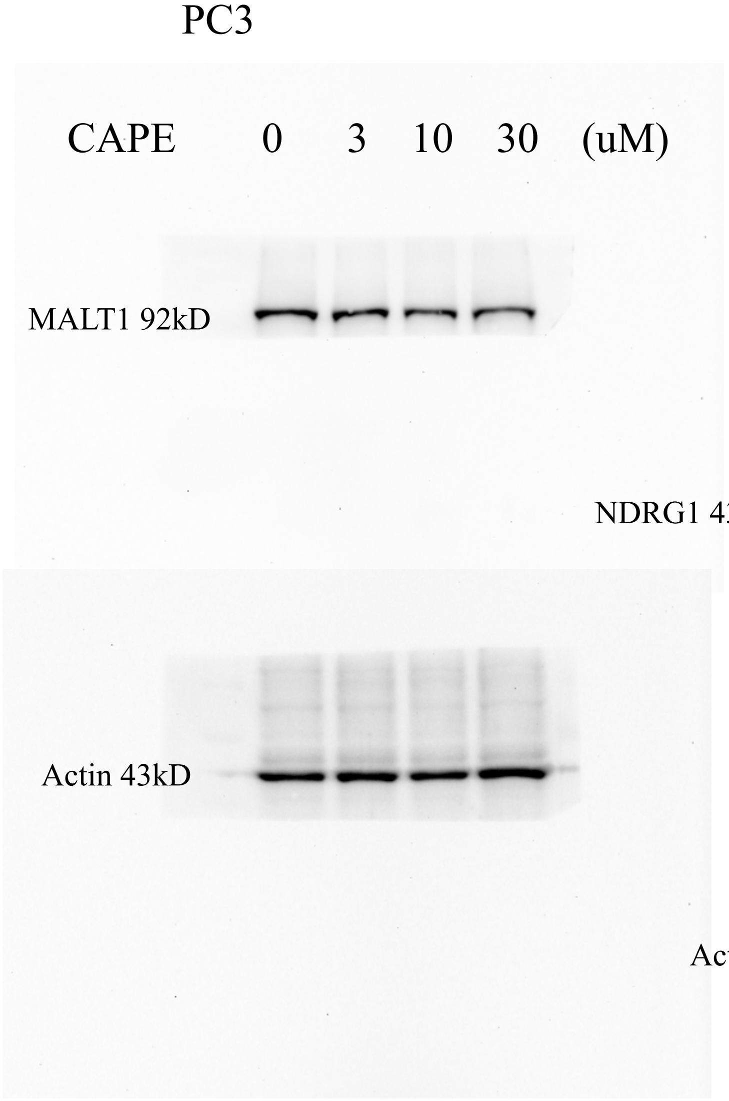
PC3
CAPE 0 3 10 30 (uM)
MALT1 92kD
Actin 43kD

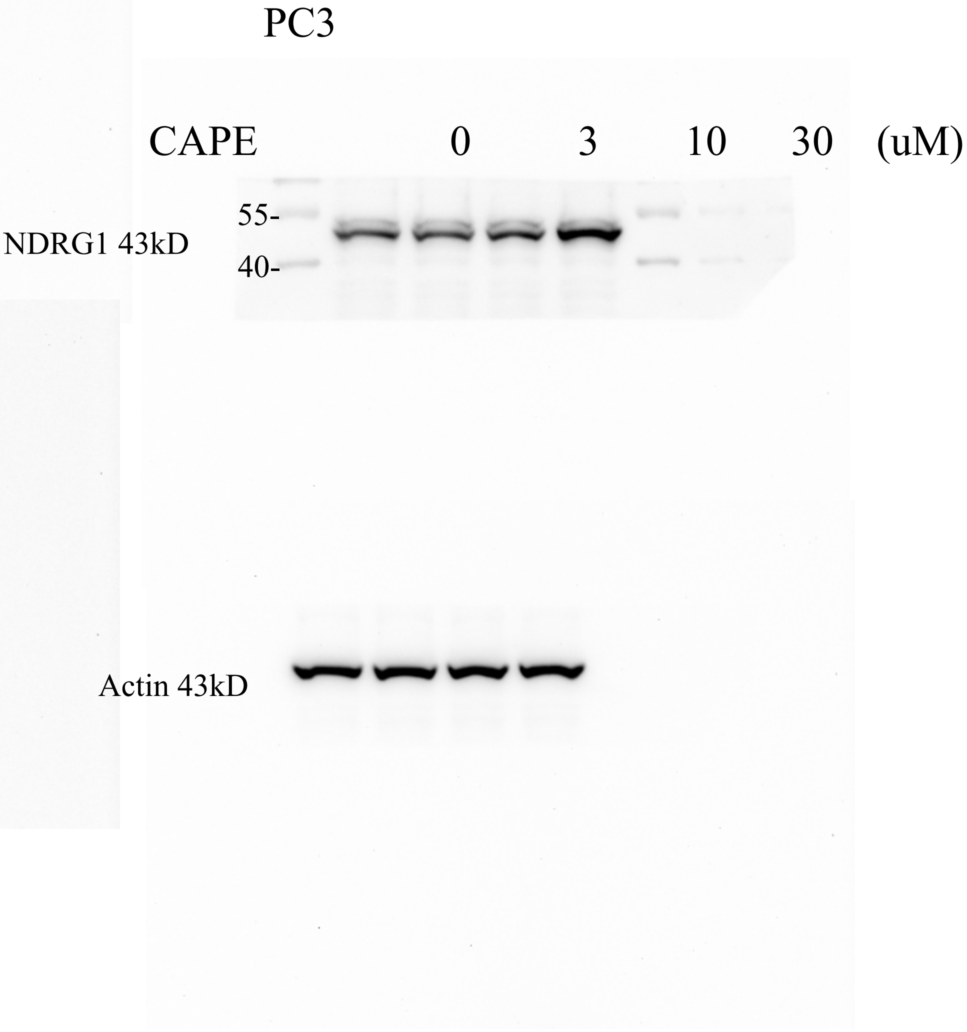
PC3
CAPE 0 3 10 30 (uM)
55-
NDRG1 43kD
40-
Actin 43kD

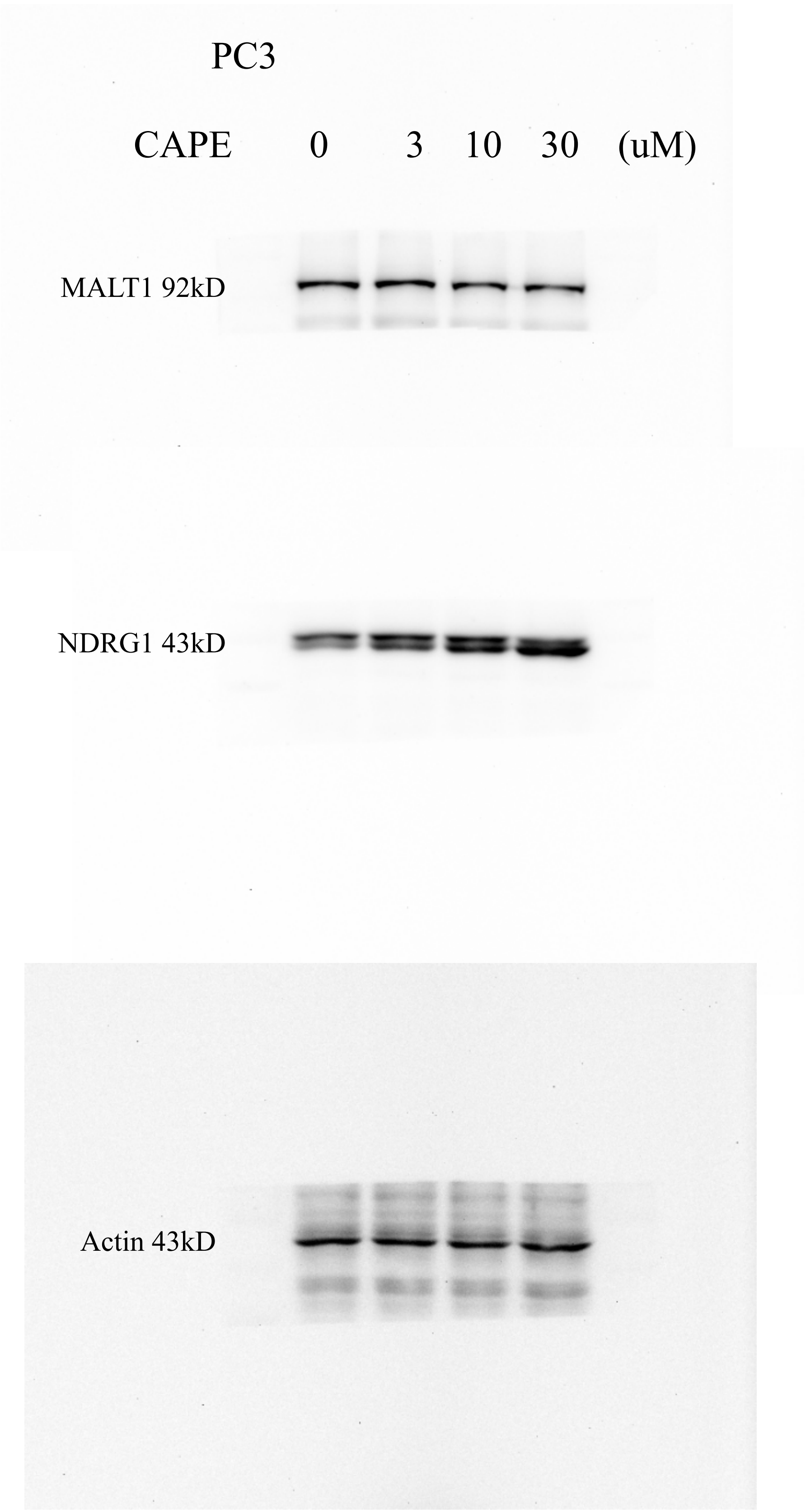
PC3
CAPE 0 3 10 30 (uM)
MALT1 92kD
NDRG1 43kD
Actin 43kD

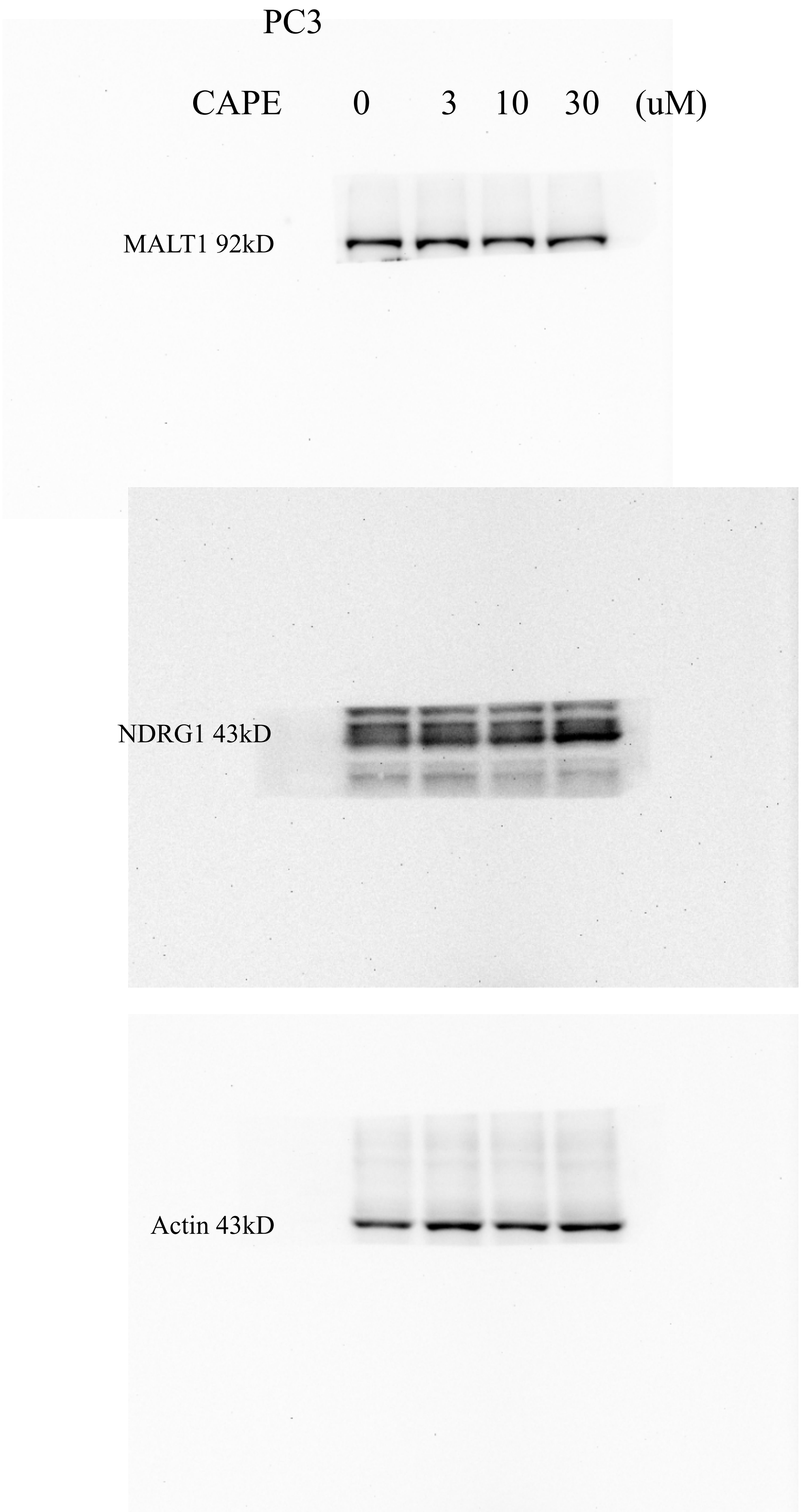
PC3
CAPE 0 3 10 30 (uM)
MALT1 92kD
NDRG1 43kD
Actin 43kD

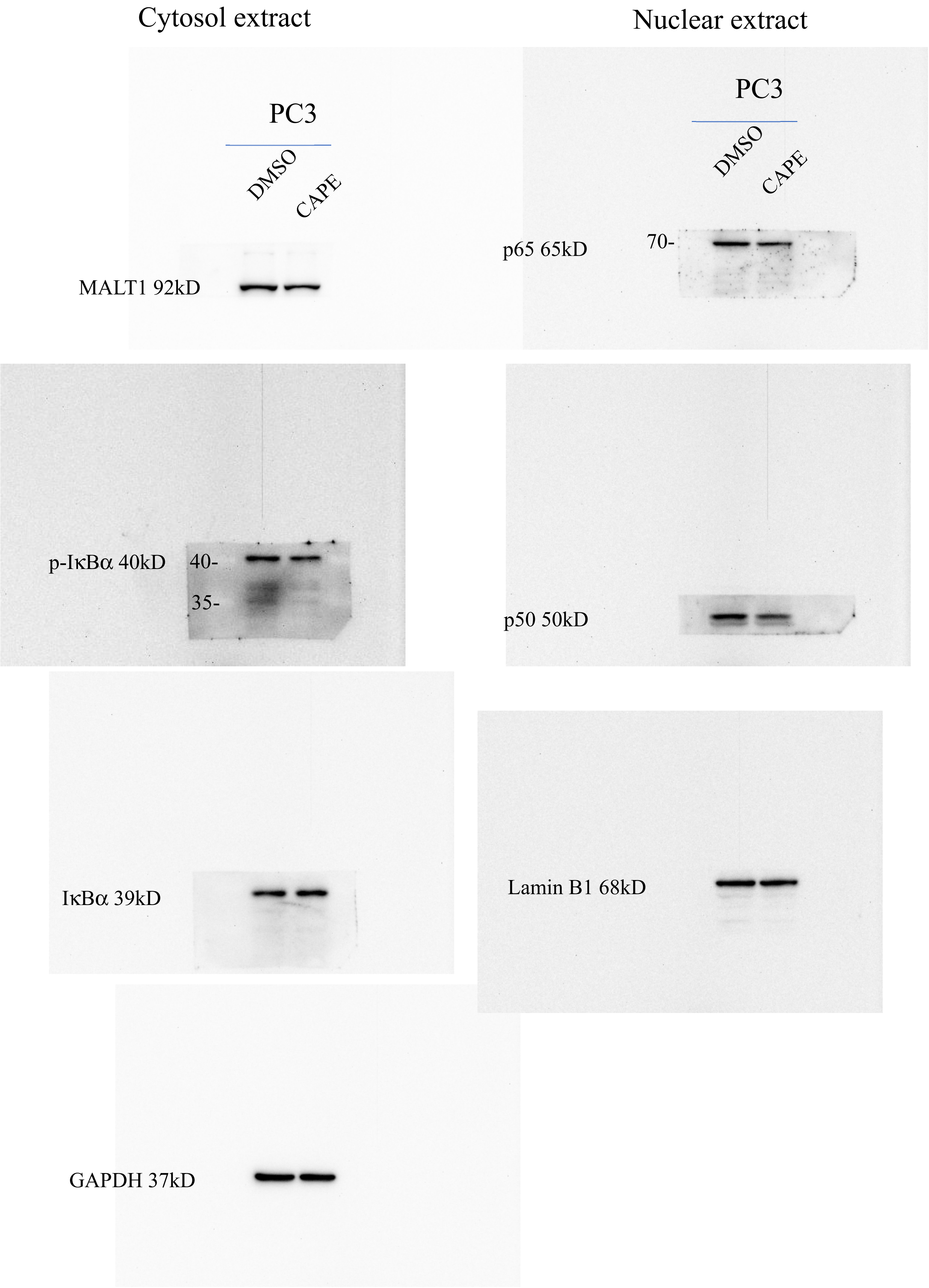
Cytosol extract
Nuclear extract
PC3
DMSO
CAPE
MALT1 92kD
PC3
DMSO
CAPE
p65 65kD
70-
p-IκBα 40kD
40-
35-
p50 50kD
IκBα 39kD
Lamin B1 68kD
GAPDH 37kD

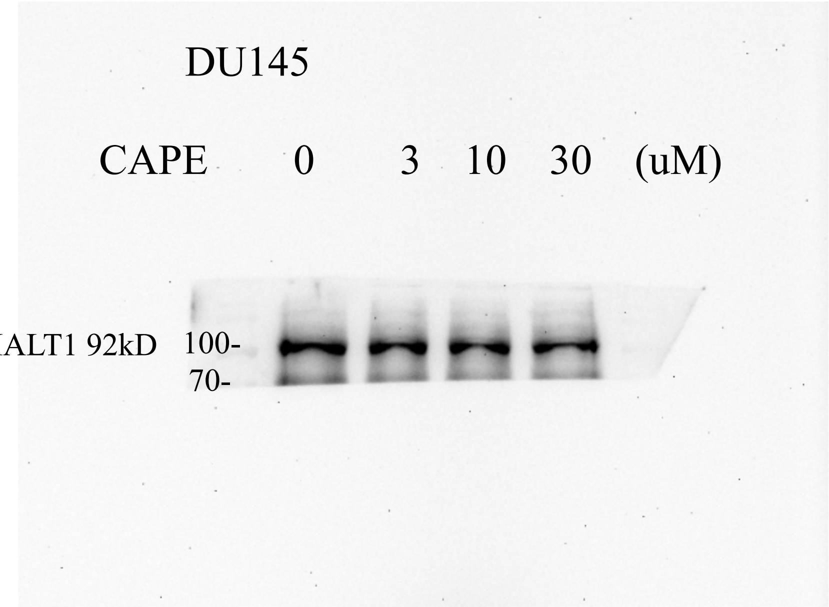
DU145
CAPE 0 3 10 30 (uM)
MALT1 92kD
100-
70-

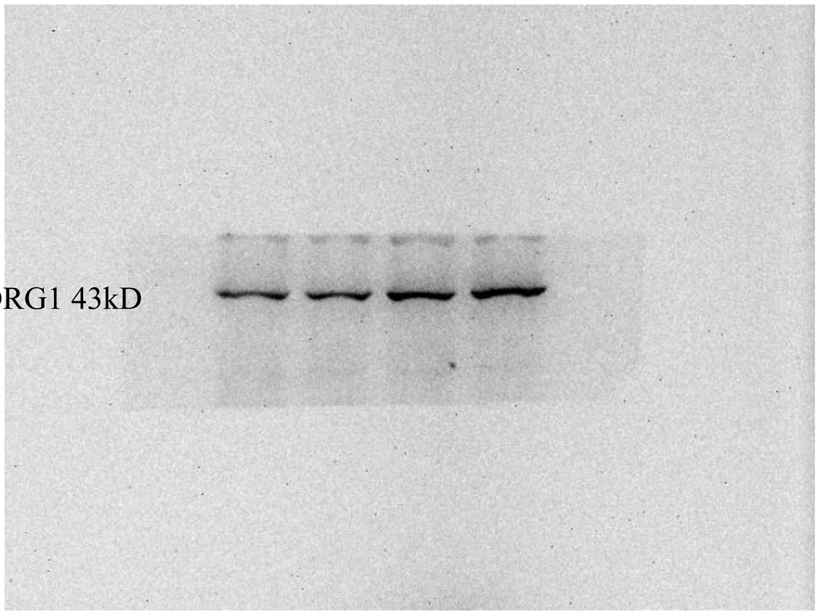
NDRG1 43kD

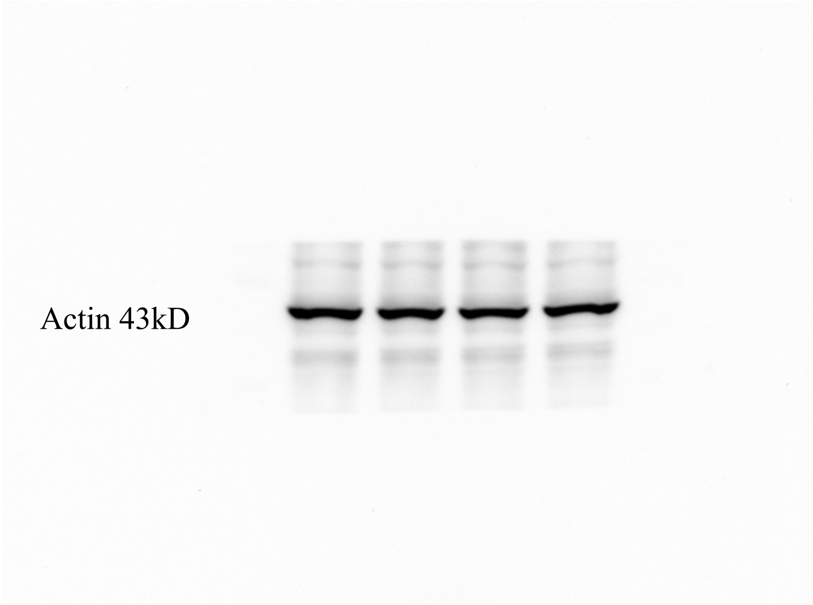
Actin 43kD

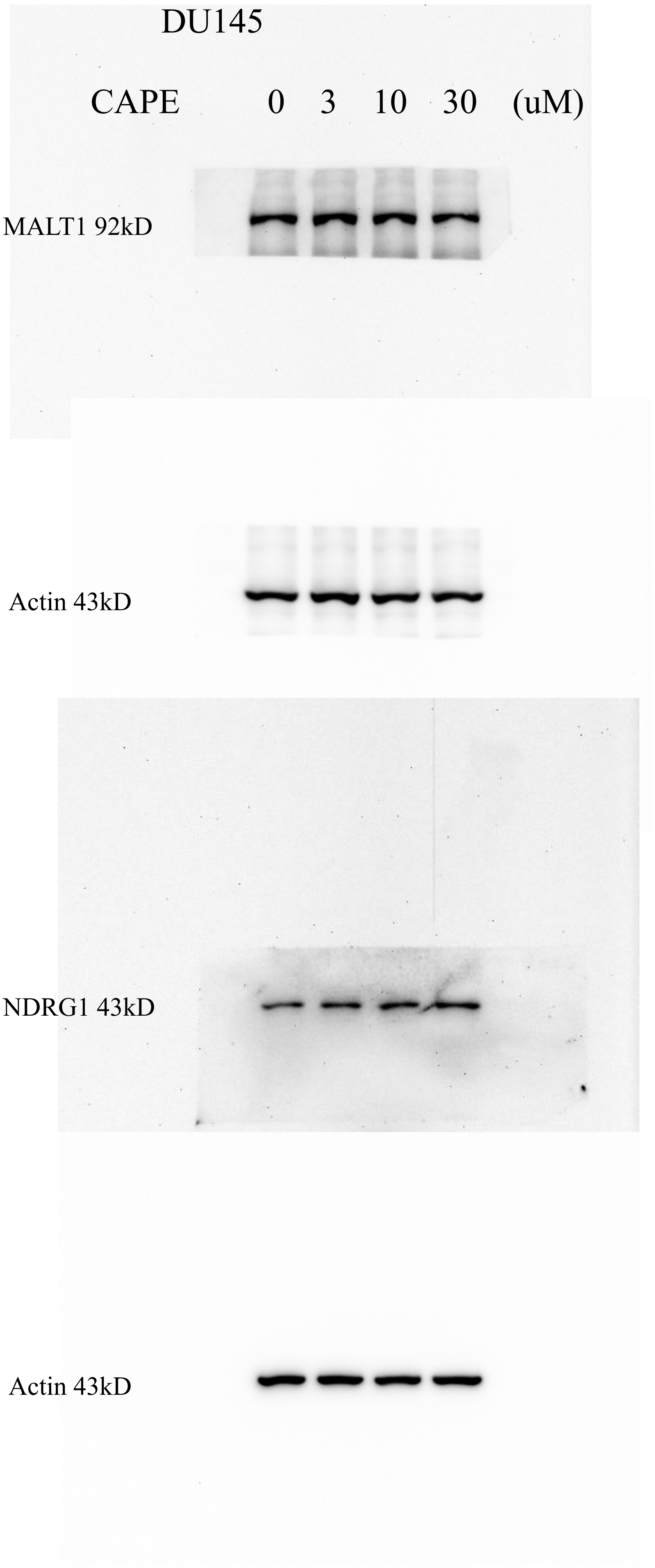
DU145
CAPE 0 3 10 30 (uM)
MALT1 92kD
Actin 43kD
NDRG1 43kD
Actin 43kD

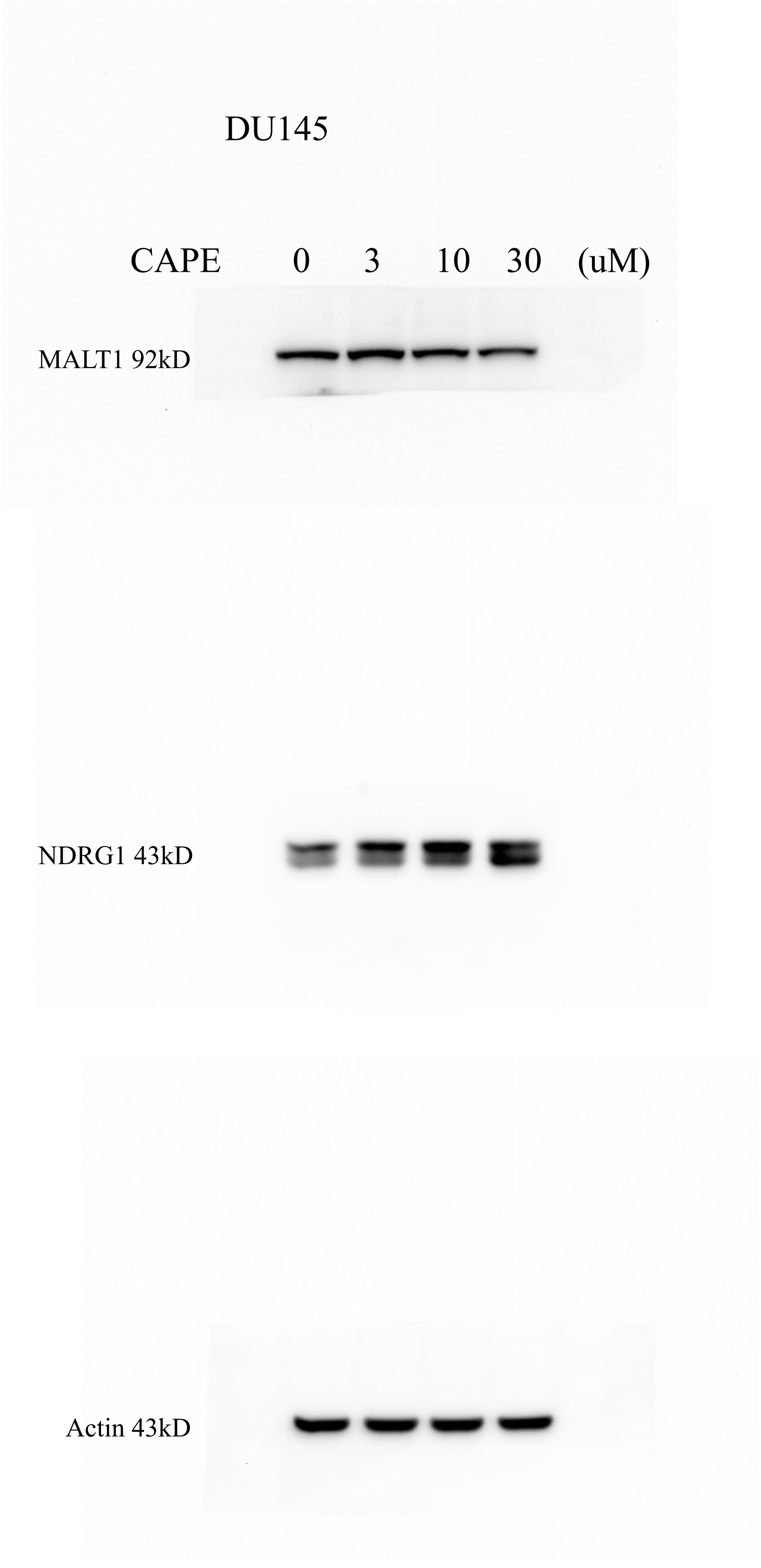

Figure S3: Original uncropped Western blots of figure 3
