# Supplementary material for: The Antitumor Effect of Caffeic Acid Phenethyl Ester by Downregulating Mucosa-Associated Lymphoid Tissue 1 via AR/p53/NF-κB Signaling in Prostate Carcinoma Cells
Source: Cancers (Basel). 2022 Jan 6;14(2):274. doi: 10.3390/cancers14020274 (PMC8773797; doi:10.3390/cancers14020274)
Supplement: Supplementary file 1 [file cancers-14-00274-s001.zip › cancers-1524042-supplementary/Figure S4.pdf]

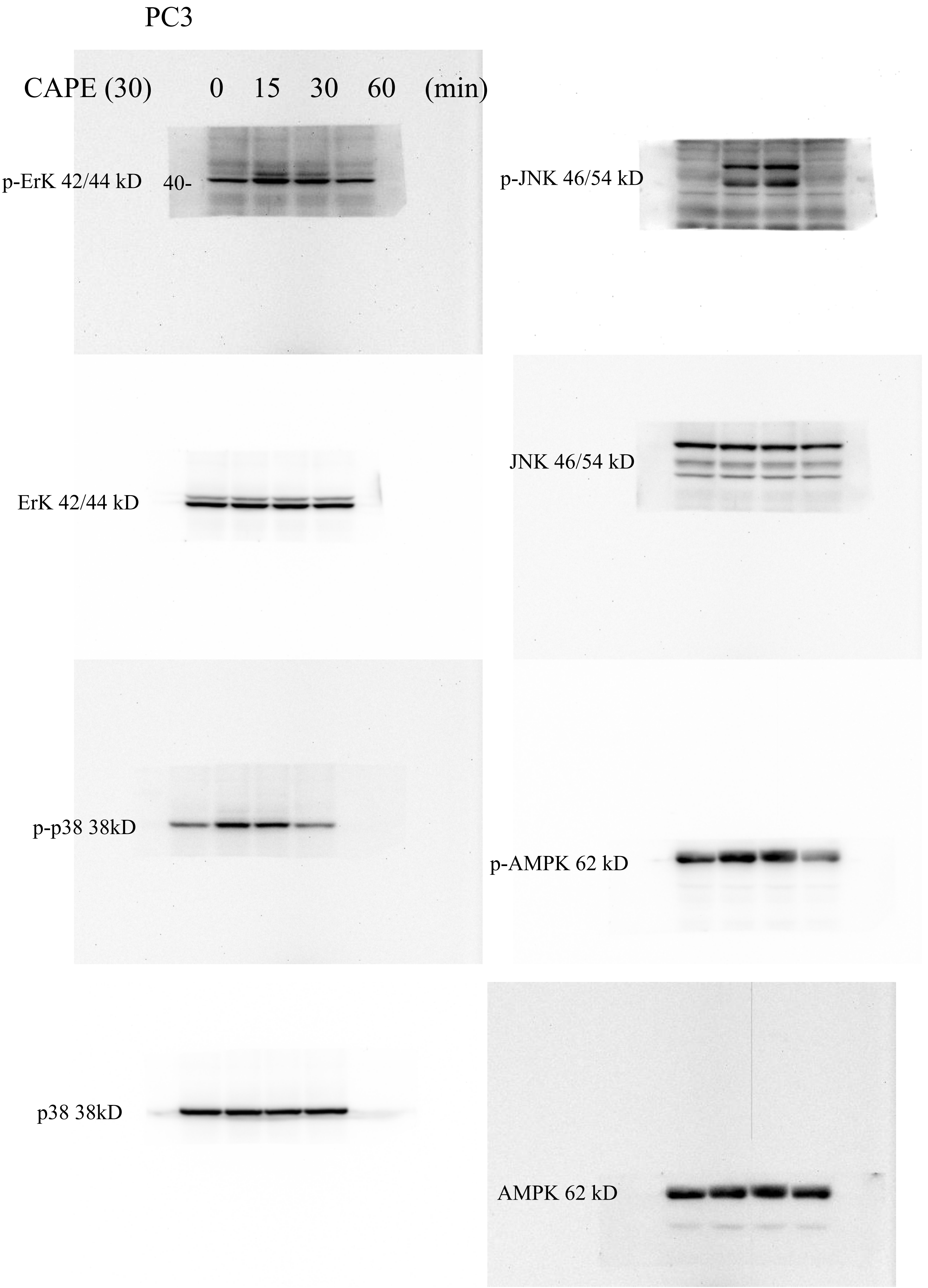
PC3
CAPE (30) 0 15 30 60 (min)
p-ErK 42/44 kD
40-
p-JNK 46/54 kD
ErK 42/44 kD
JNK 46/54 kD
p-p38 38kD
p-AMPK 62 kD
p38 38kD
AMPK 62 kD

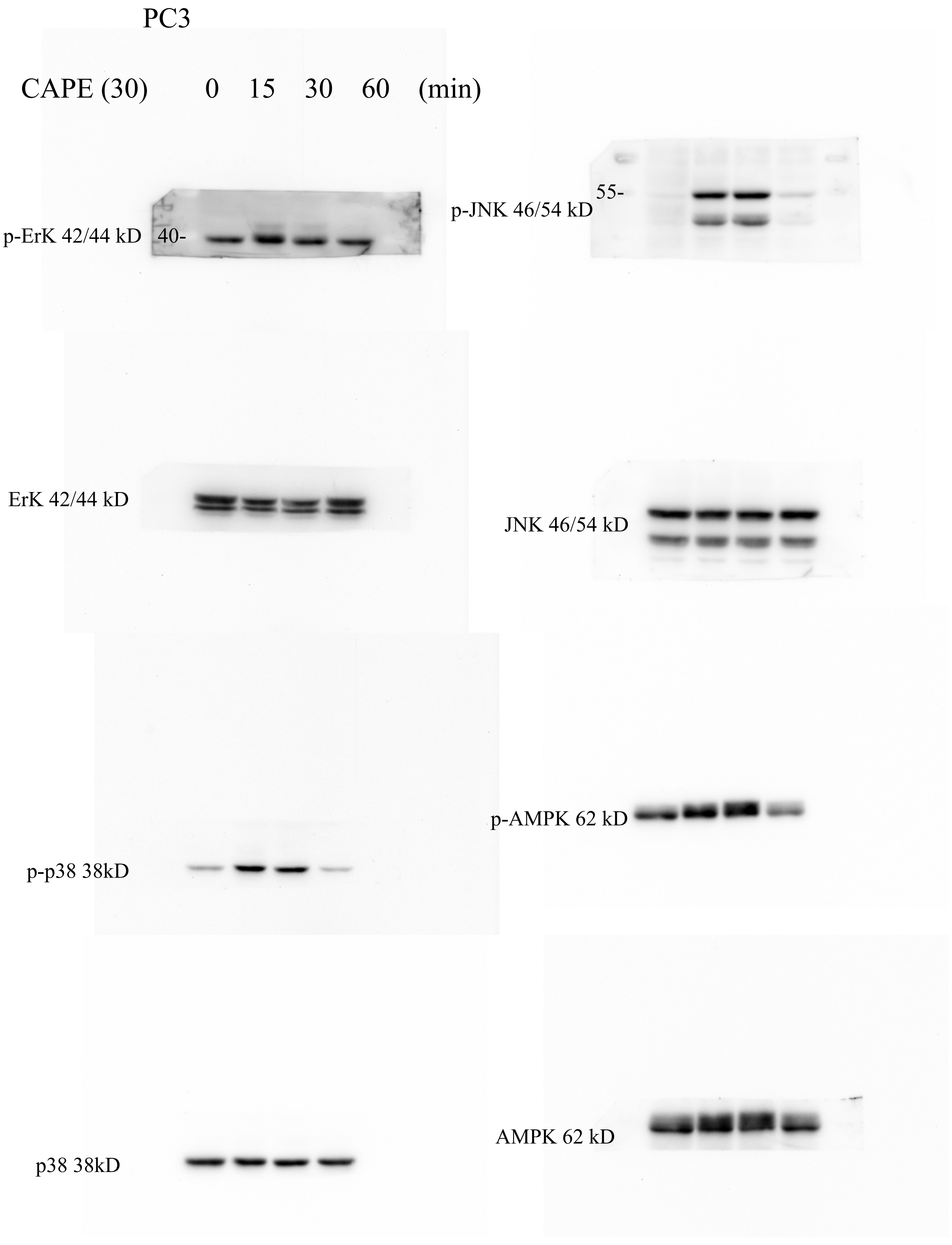
PC3
CAPE (30) 0 15 30 60 (min)
p-ErK 42/44 kD 40-
p-JNK 46/54 kD 55-
ErK 42/44 kD
JNK 46/54 kD
p-AMPK 62 kD
p-p38 38kD
AMPK 62 kD
p38 38kD

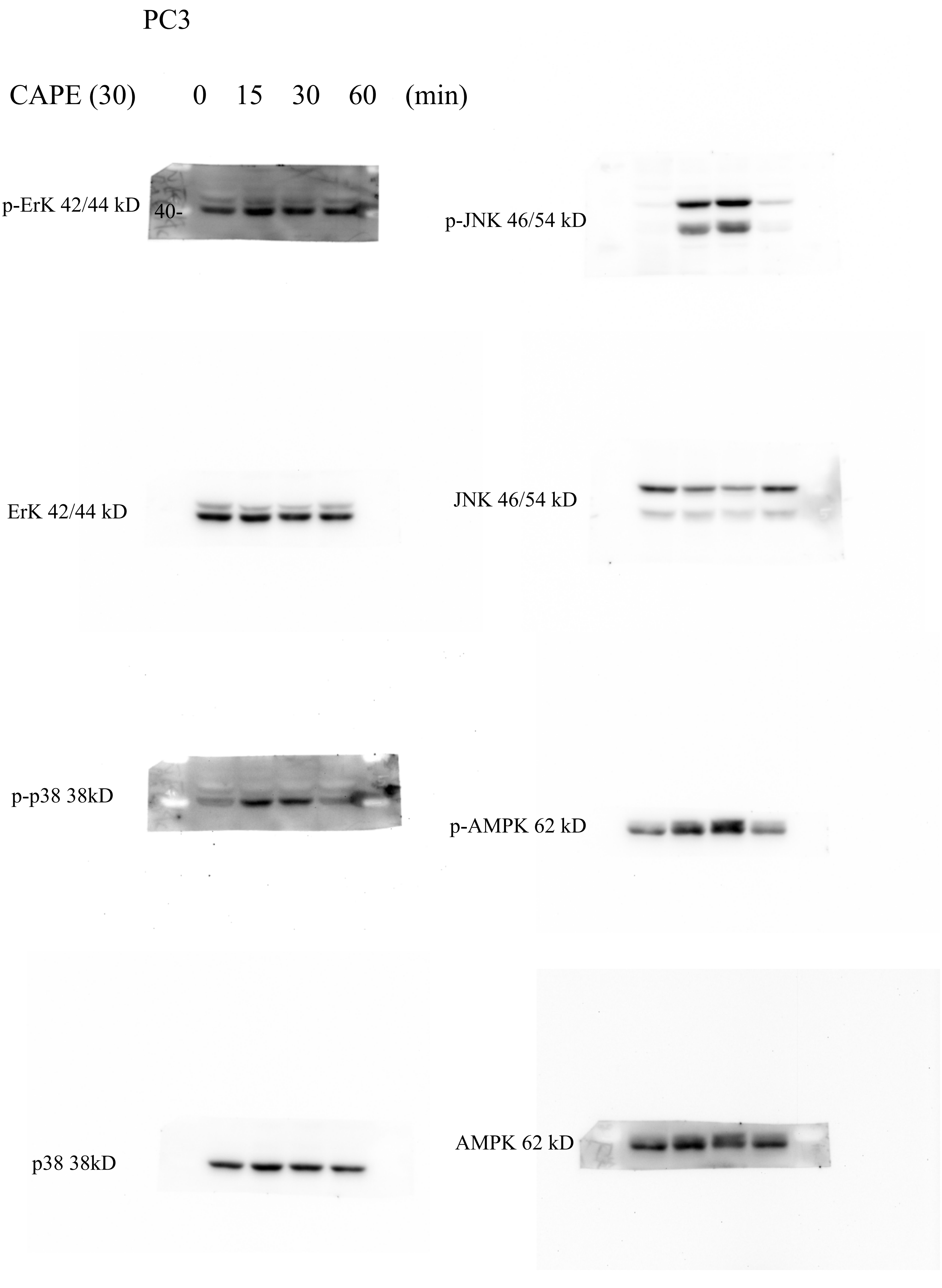
PC3
CAPE (30) 0 15 30 60 (min)
p-ErK 42/44 kD
40-
p-JNK 46/54 kD
ErK 42/44 kD
JNK 46/54 kD
p-p38 38kD
p-AMPK 62 kD
p38 38kD
AMPK 62 kD

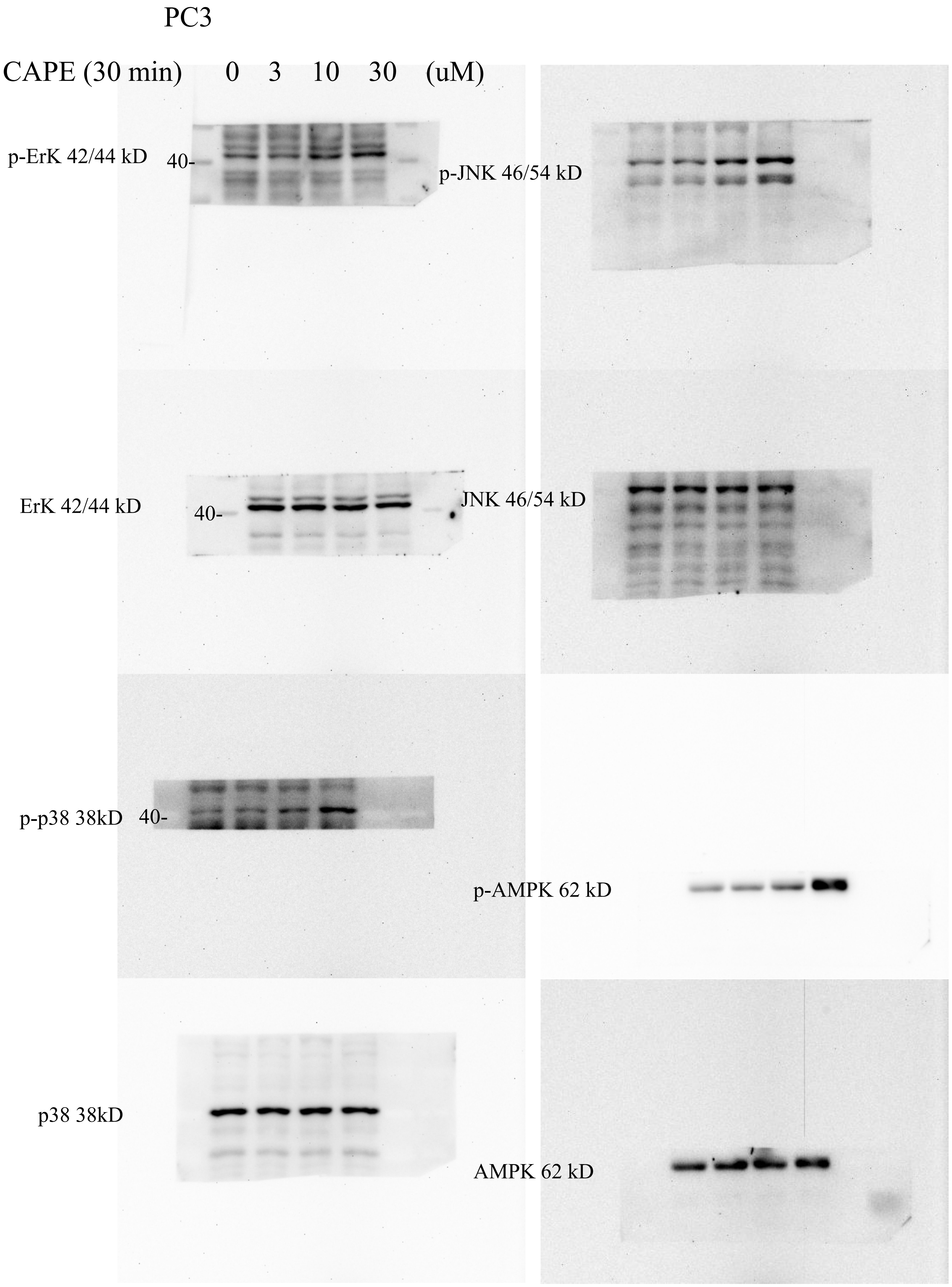
PC3
CAPE (30 min) 0 3 10 30 (uM)
p-ErK 42/44 kD
40-
p-JNK 46/54 kD
ErK 42/44 kD
40-
JNK 46/54 kD
p-p38 38kD
40-
p-AMPK 62 kD
p38 38kD
AMPK 62 kD

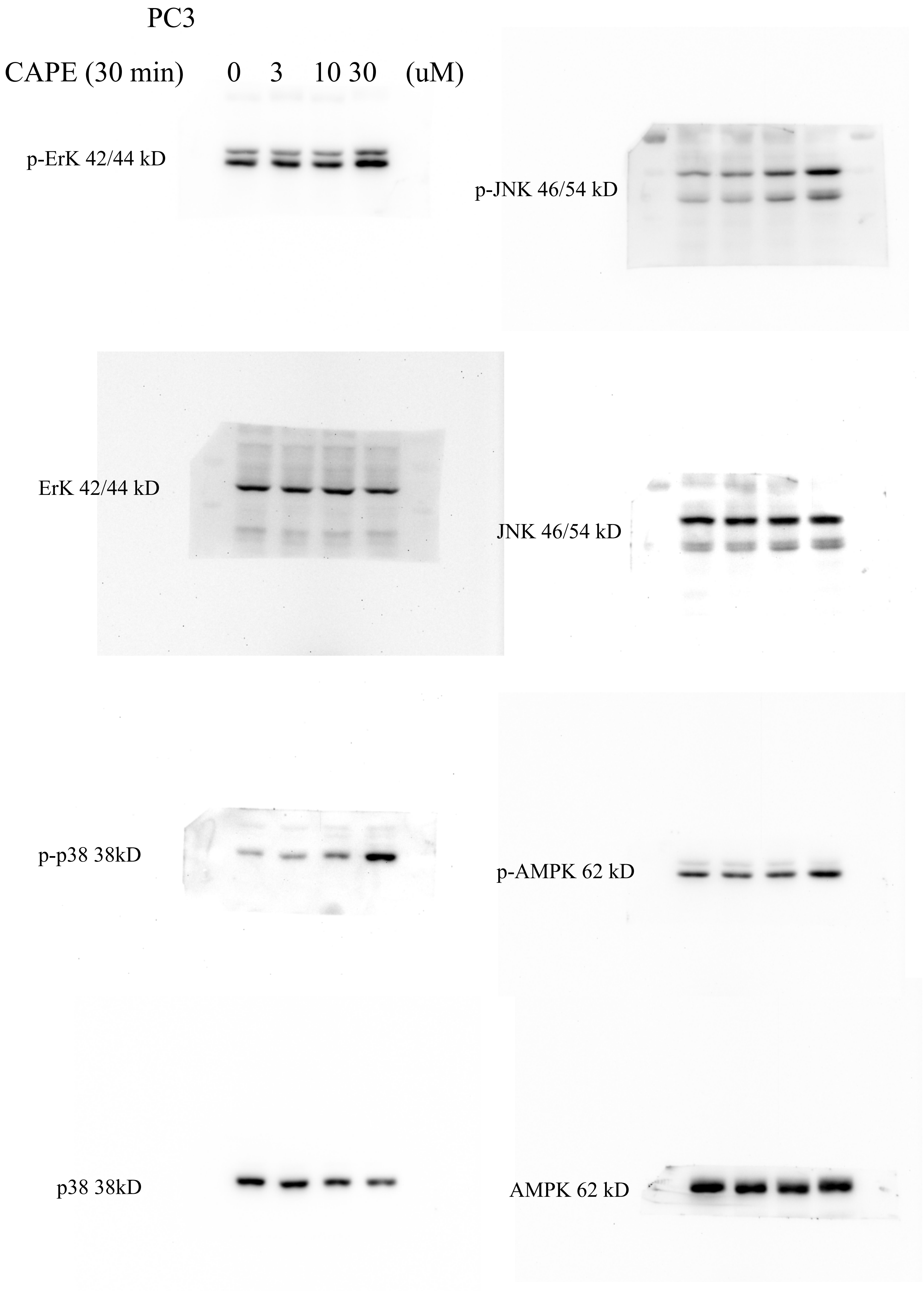
PC3
CAPE (30 min) 0 3 10 30 (uM)
p-ErK 42/44 kD
p-JNK 46/54 kD
ErK 42/44 kD
JNK 46/54 kD
p-p38 38kD
p-AMPK 62 kD
p38 38kD
AMPK 62 kD

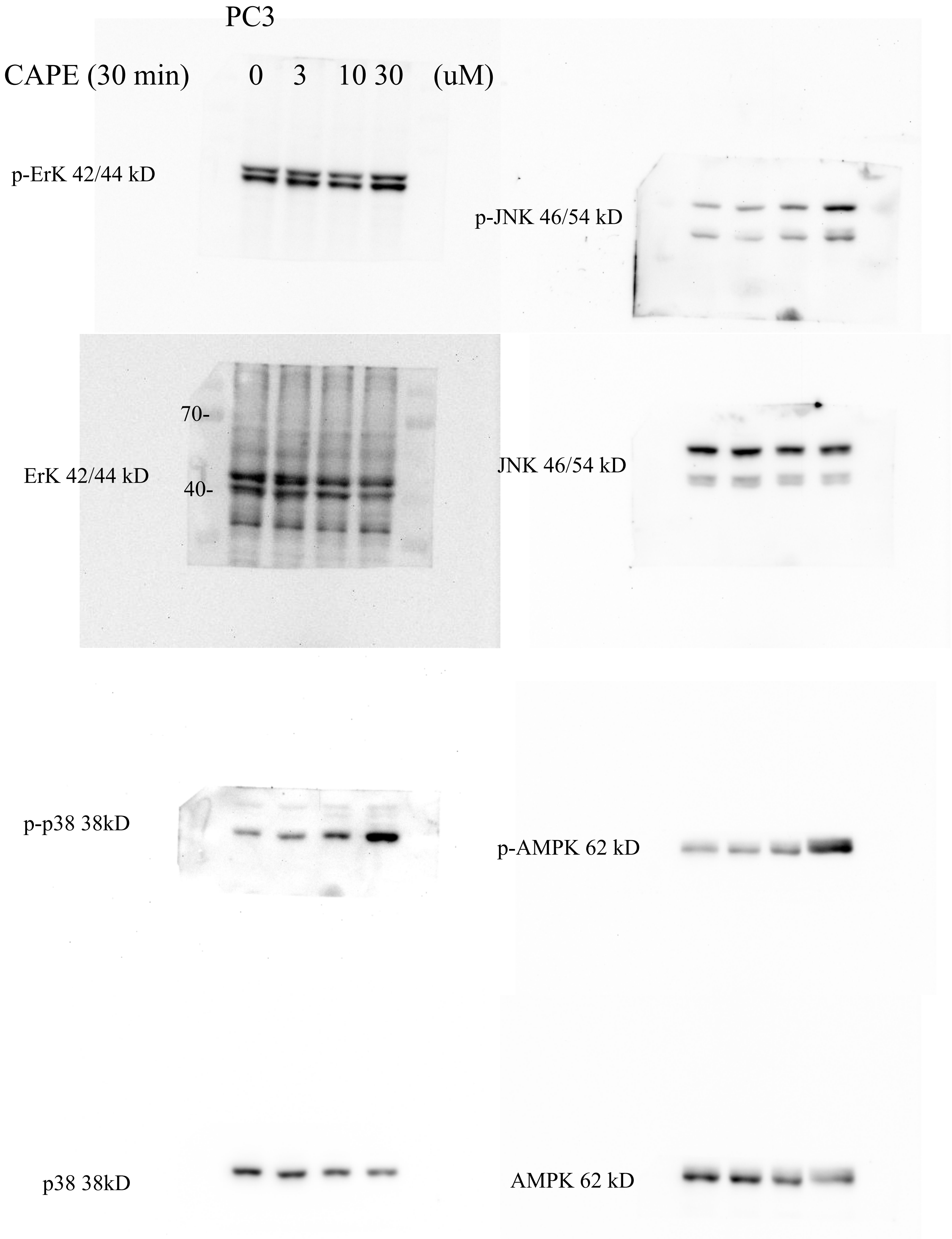

Figure S4: Original uncropped Western blots of figure 5
