# Supplementary material for: The Antitumor Effect of Caffeic Acid Phenethyl Ester by Downregulating Mucosa-Associated Lymphoid Tissue 1 via AR/p53/NF-κB Signaling in Prostate Carcinoma Cells
Source: Cancers (Basel). 2022 Jan 6;14(2):274. doi: 10.3390/cancers14020274 (PMC8773797; doi:10.3390/cancers14020274)
Supplement: Supplementary file 1 [file cancers-14-00274-s001.zip › cancers-1524042-supplementary/Figure S5.pdf]

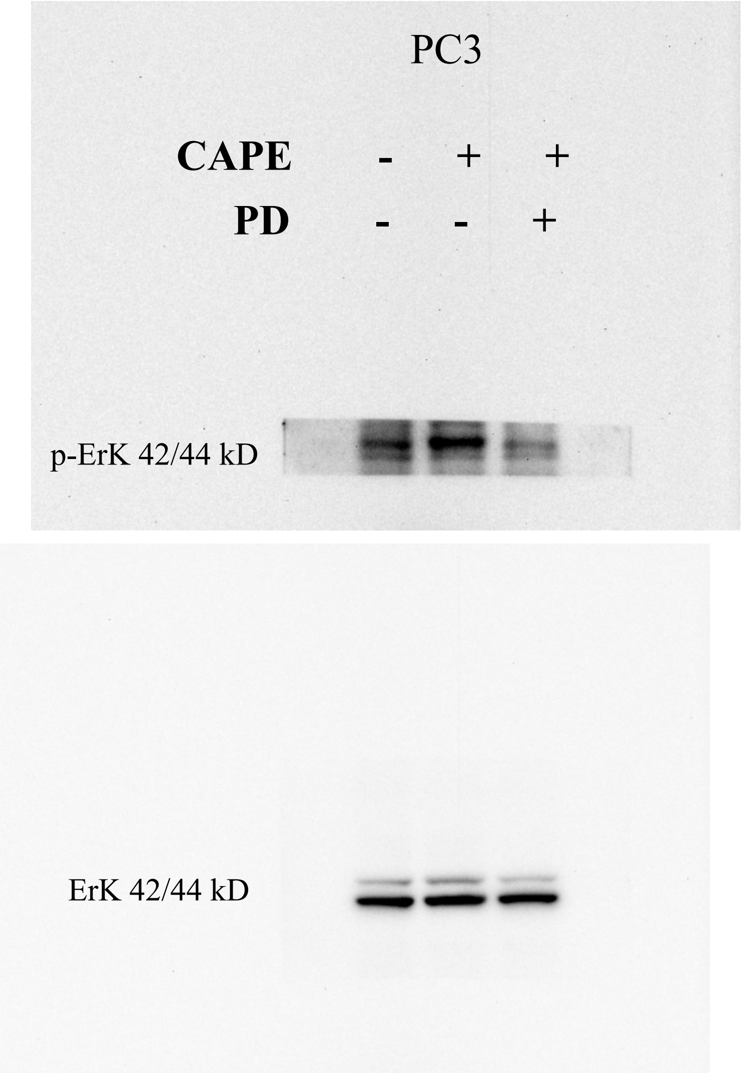
PC3
CAPE - + +
PD - - +
p-ErK 42/44 kD
ErK 42/44 kD

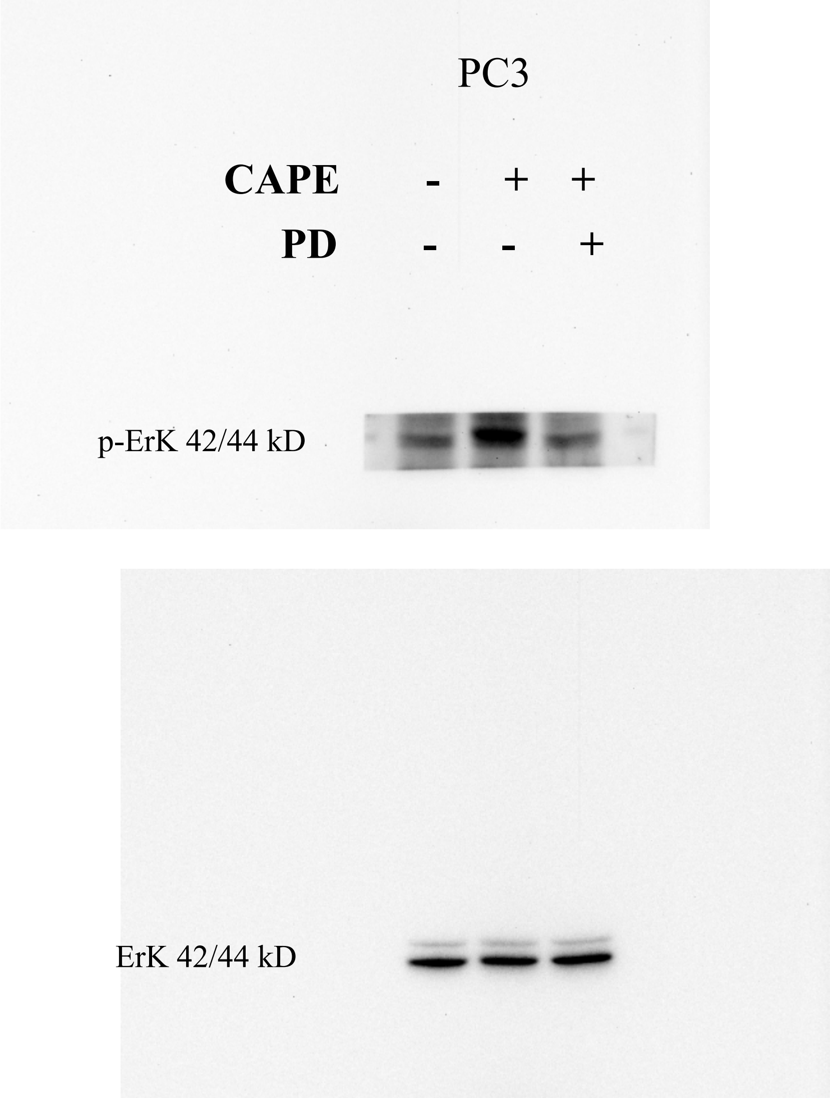
PC3
CAPE - + +
PD - - +
p-ErK 42/44 kD
ErK 42/44 kD

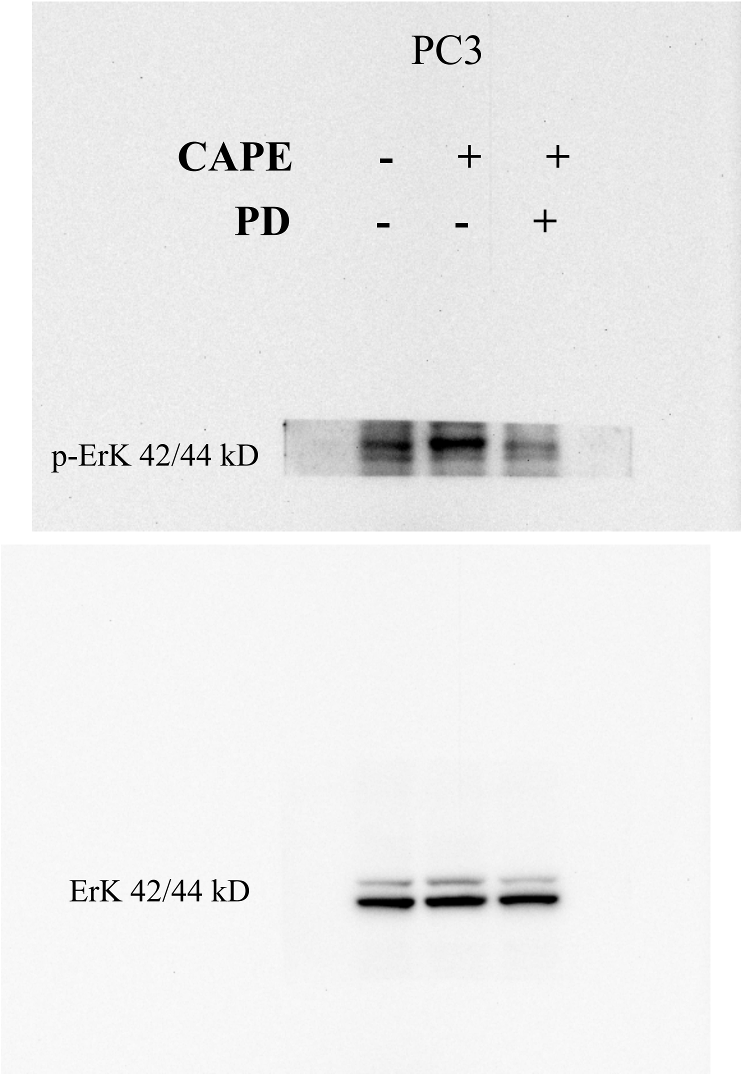
PC3
CAPE - + +
PD - - +
p-ErK 42/44 kD
ErK 42/44 kD

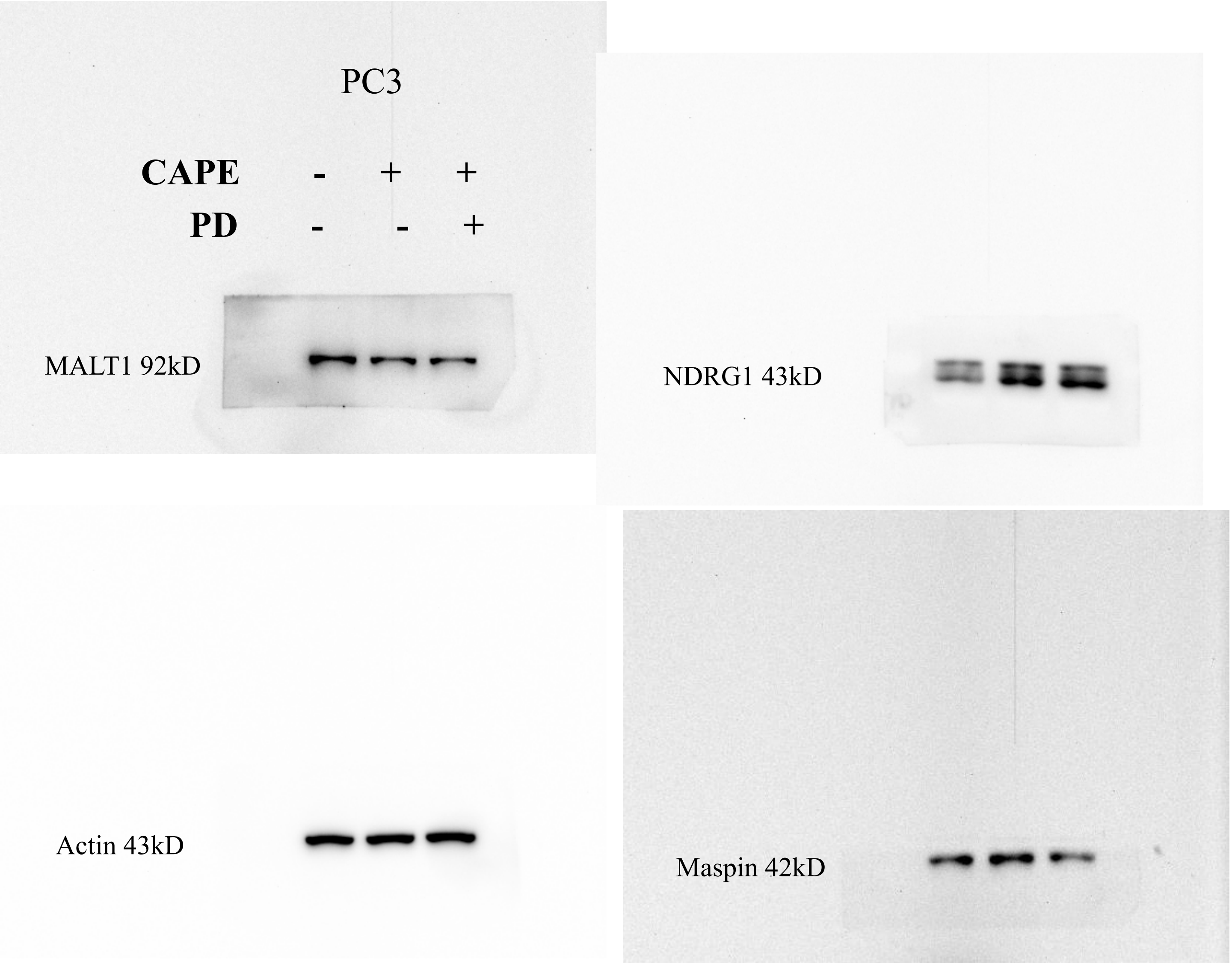
PC3
CAPE - + +
PD - - +
MALT1 92kD
NDRG1 43kD
Actin 43kD
Maspin 42kD

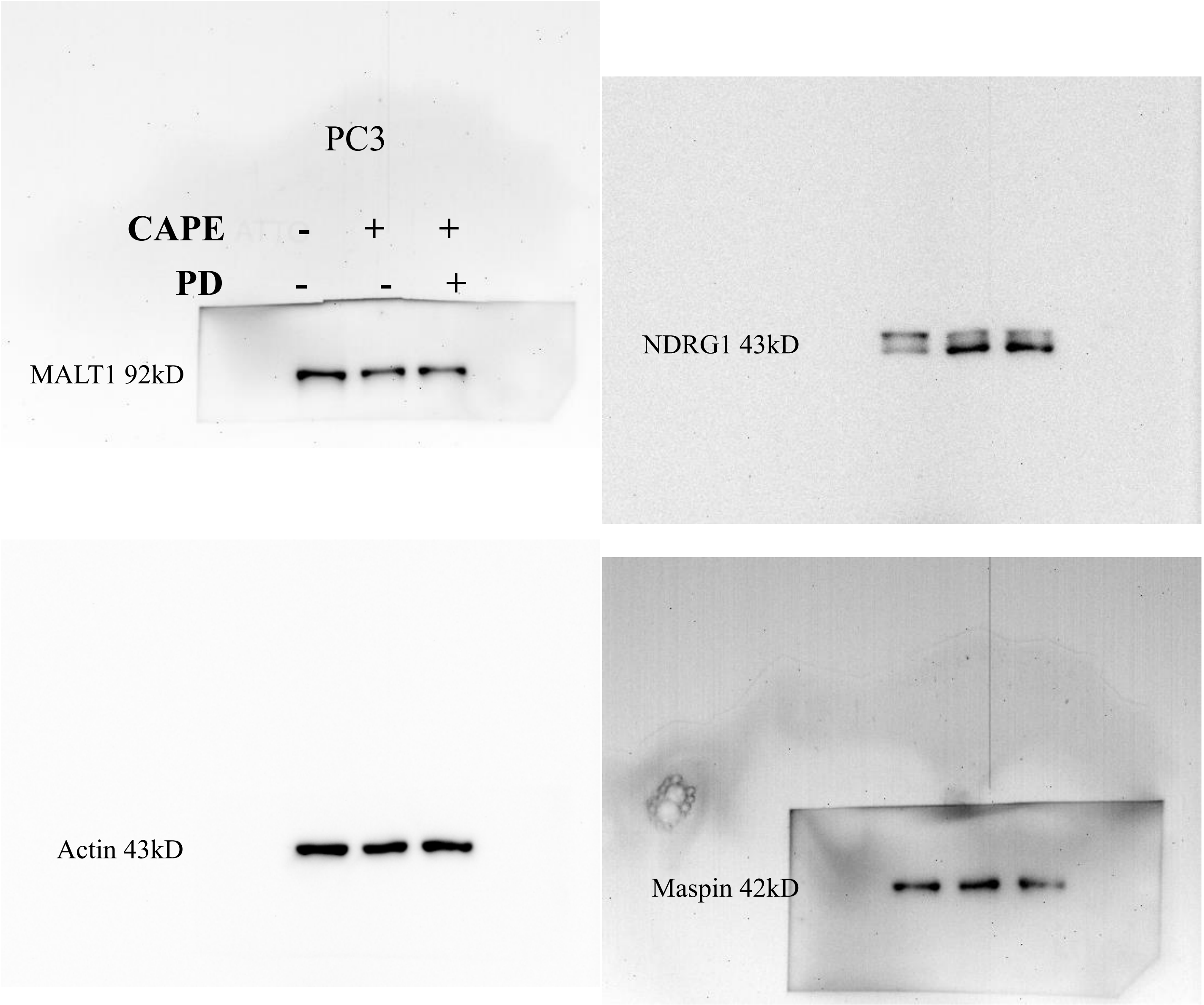
PC3
CAPE - + +
PD - - +
MALT1 92kD
NDRG1 43kD
Actin 43kD
Maspin 42kD

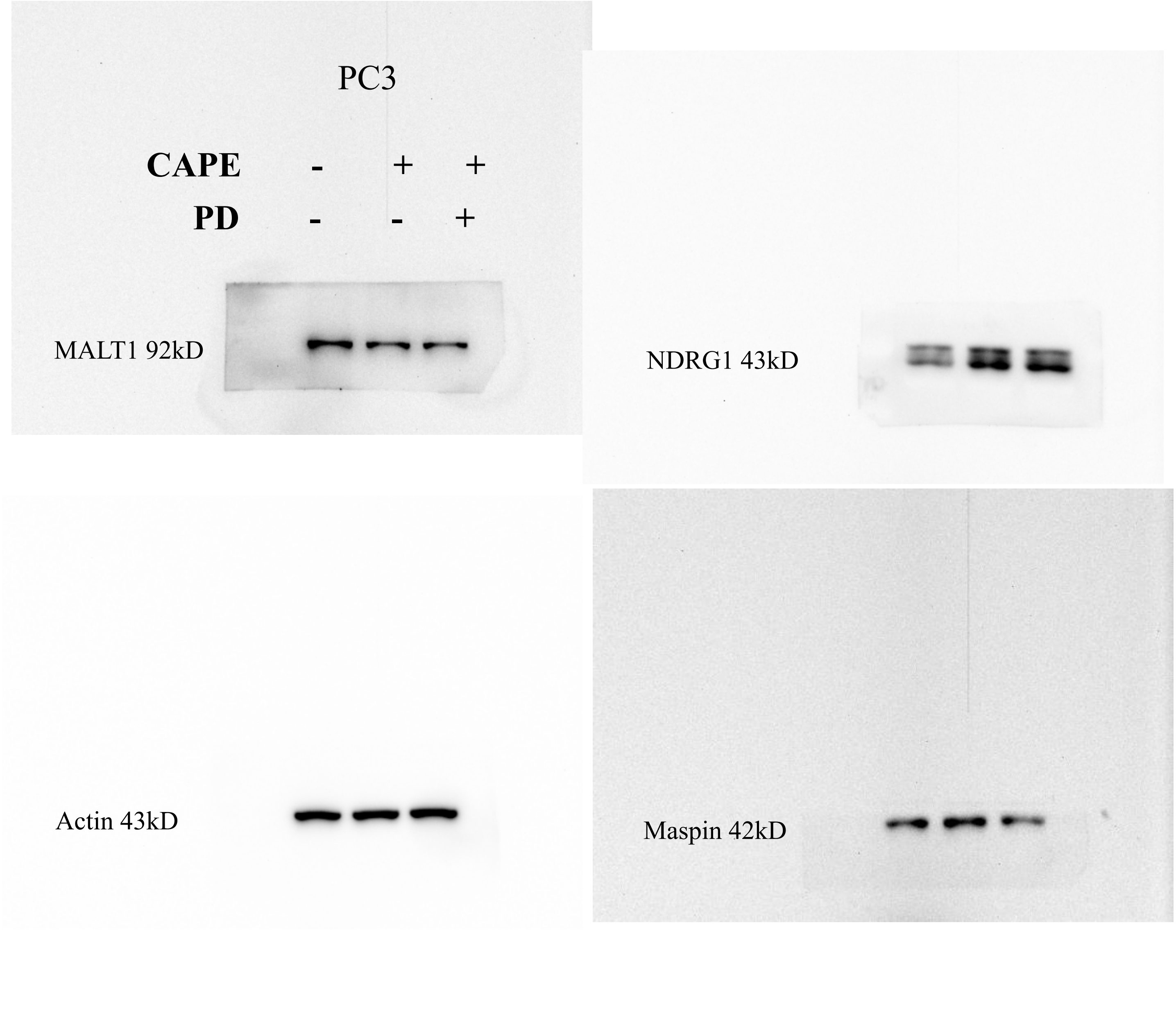
PC3
CAPE - + +
PD - - +
MALT1 92kD
NDRG1 43kD
Actin 43kD
Maspin 42kD

PC3

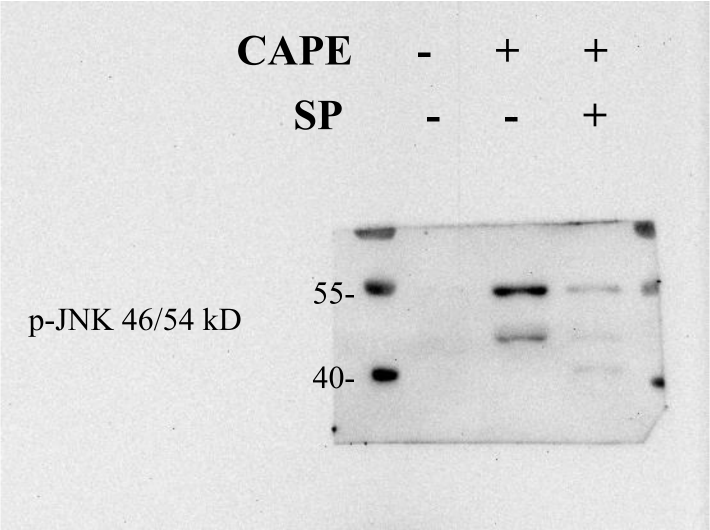


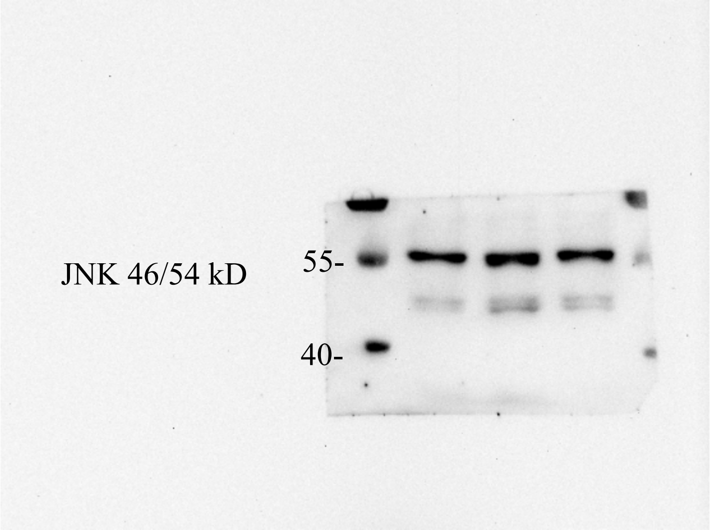

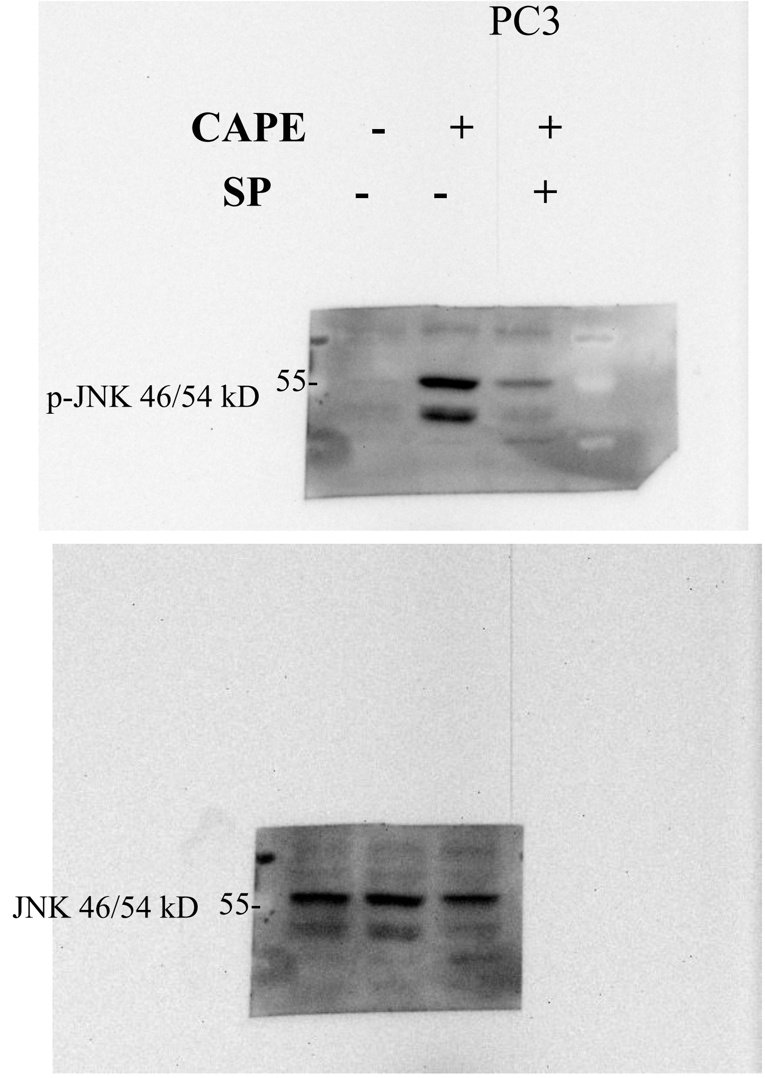
PC3
CAPE - + +
SP - - +
p-JNK 46/54 kD
55-
JNK 46/54 kD
55-

PC3

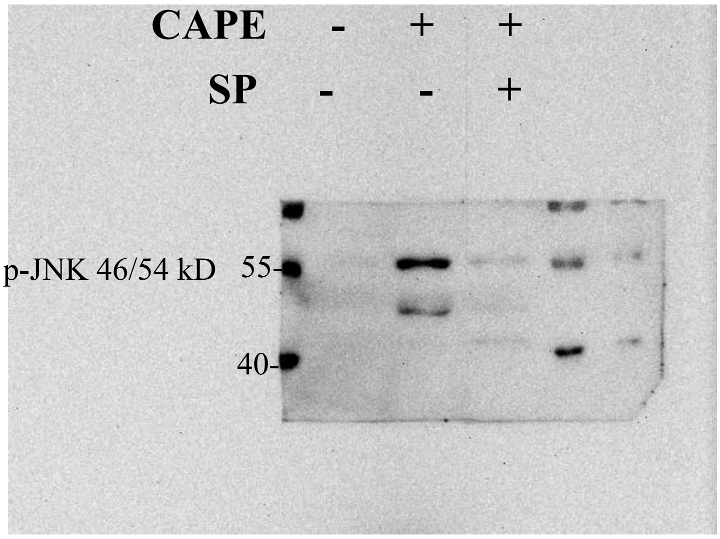


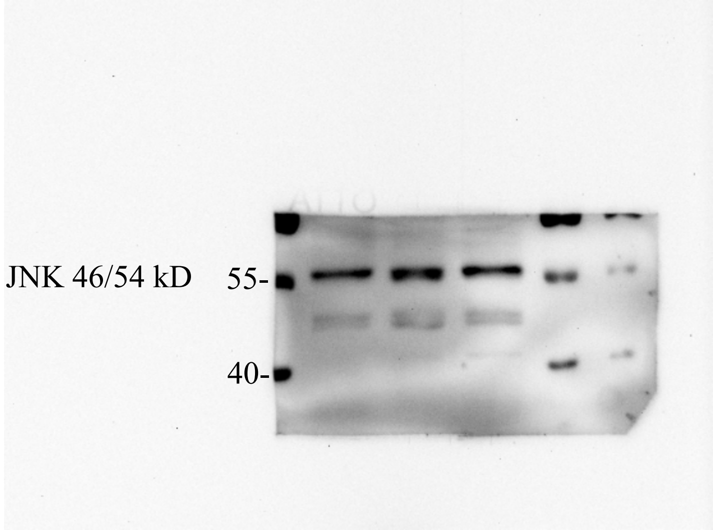

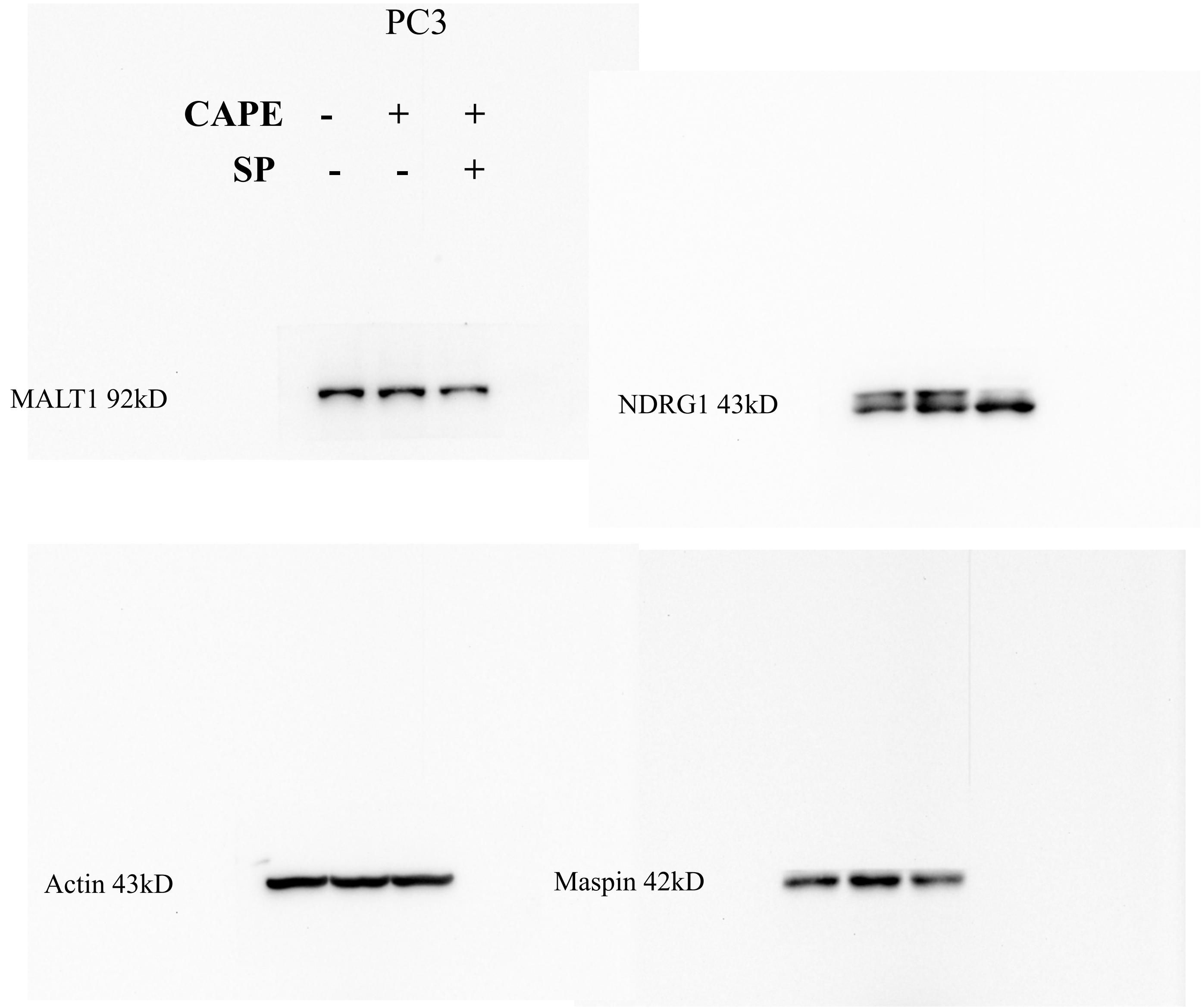
PC3
CAPE - + +
SP - - +
MALT1 92kD
NDRG1 43kD
Actin 43kD
Maspin 42kD

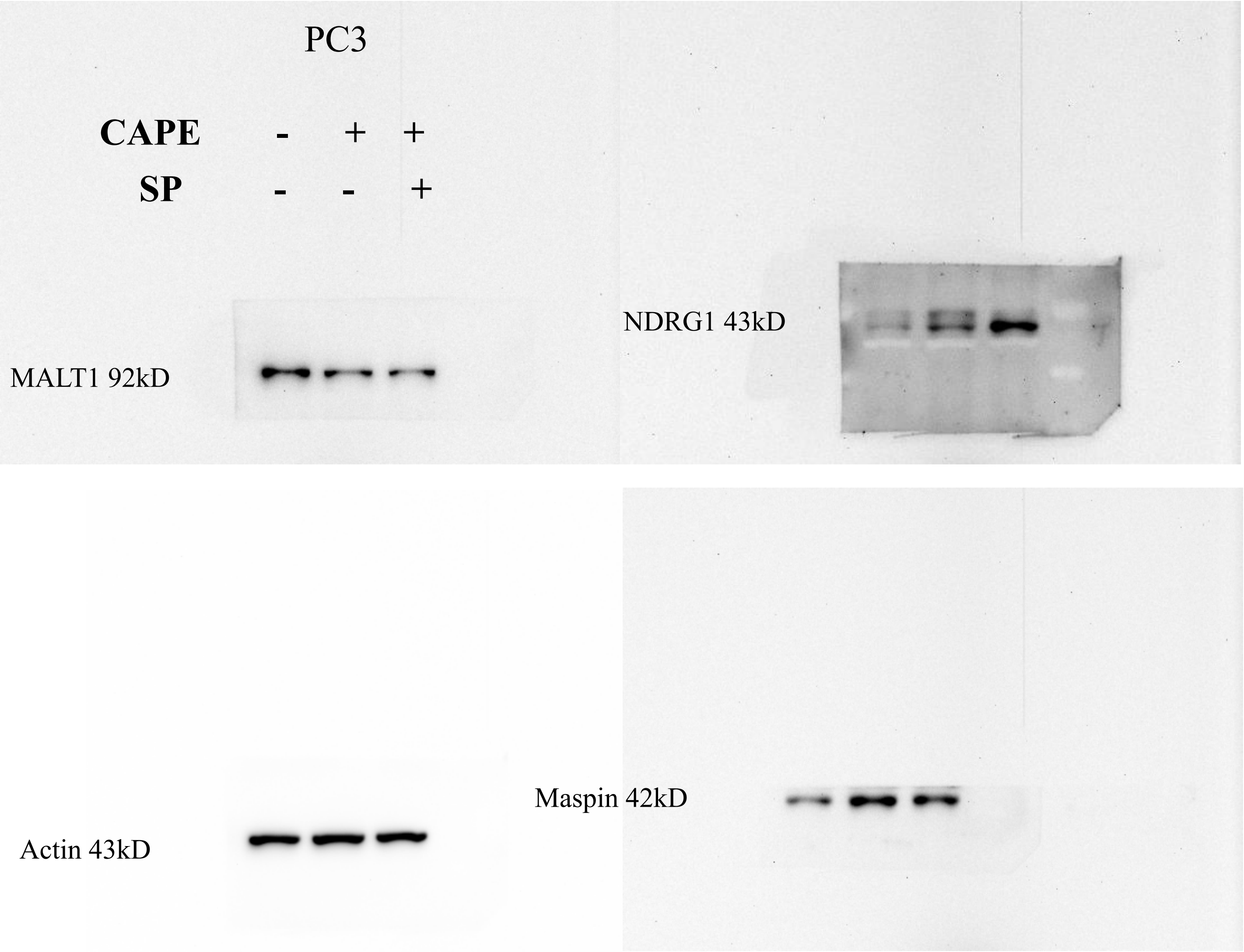
PC3
CAPE - + +
SP - - +
MALT1 92kD
NDRG1 43kD
Actin 43kD
Maspin 42kD

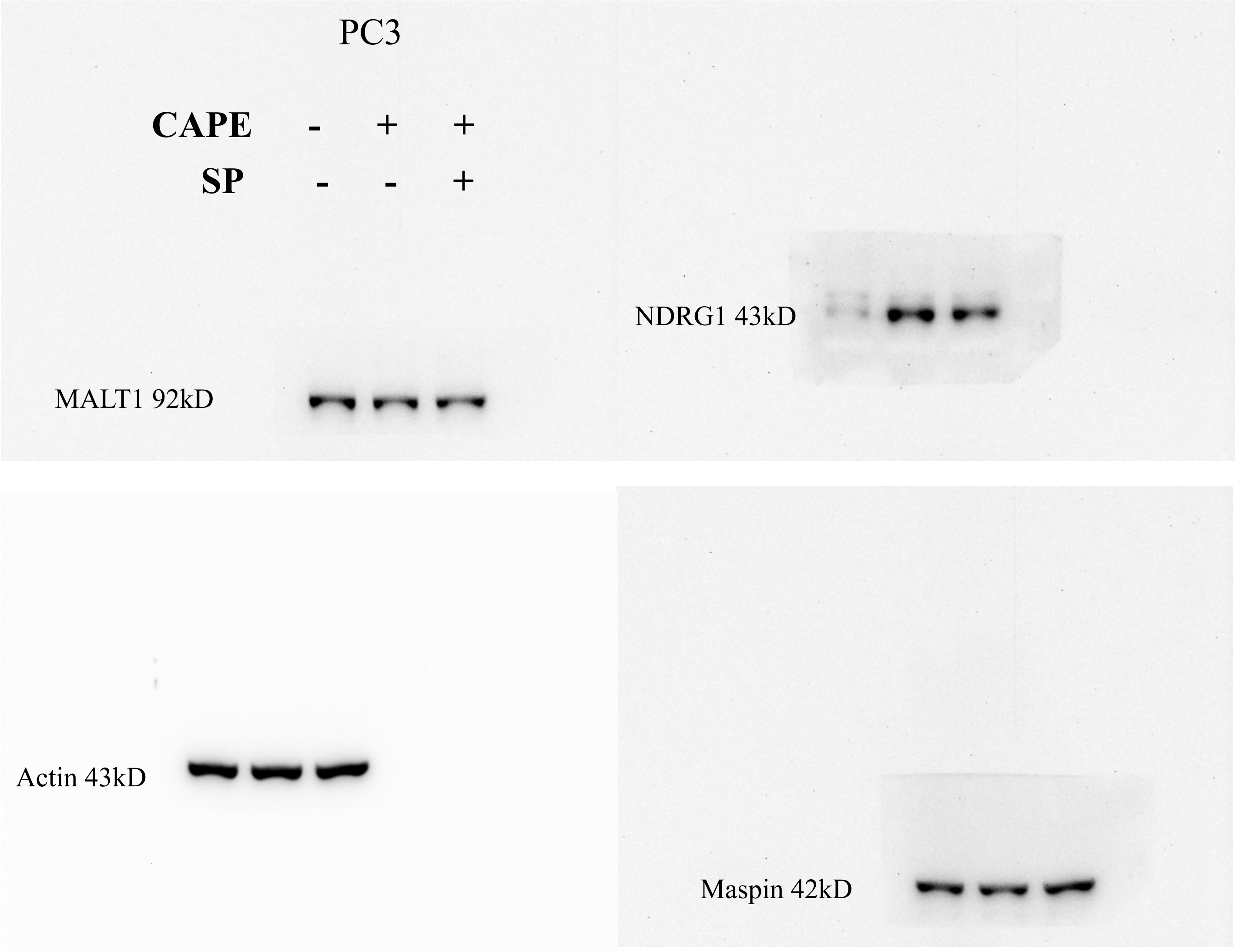
PC3
CAPE - + +
SP - - +
MALT1 92kD
NDRG1 43kD
Actin 43kD
Maspin 42kD

PC3

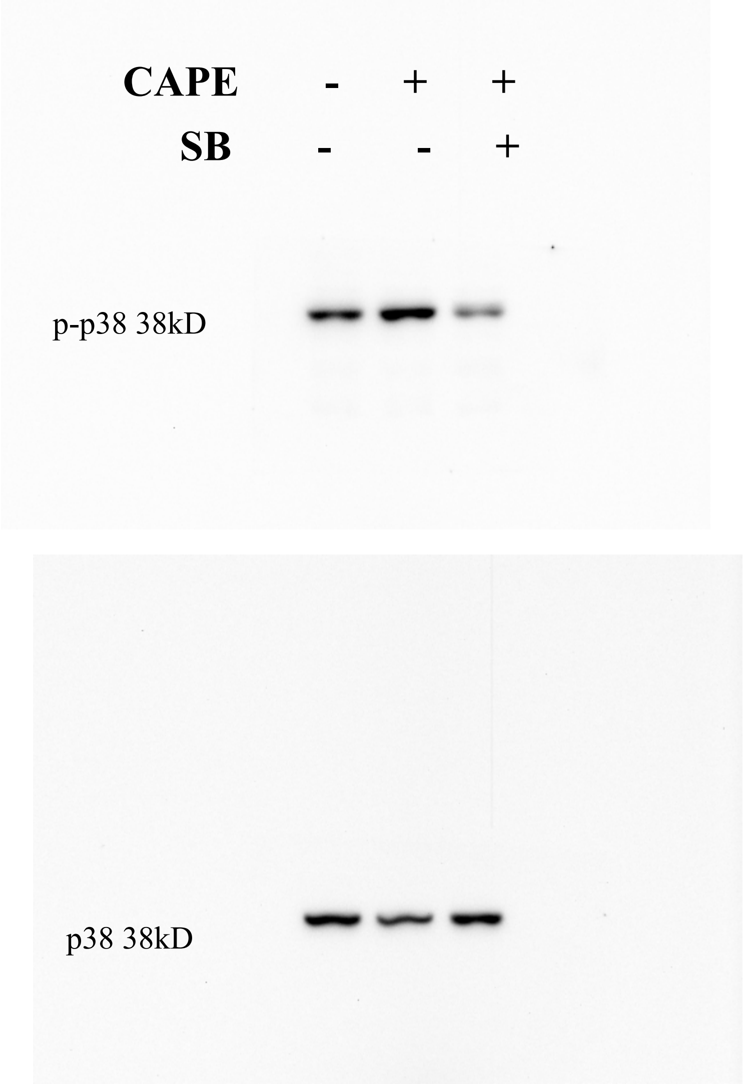

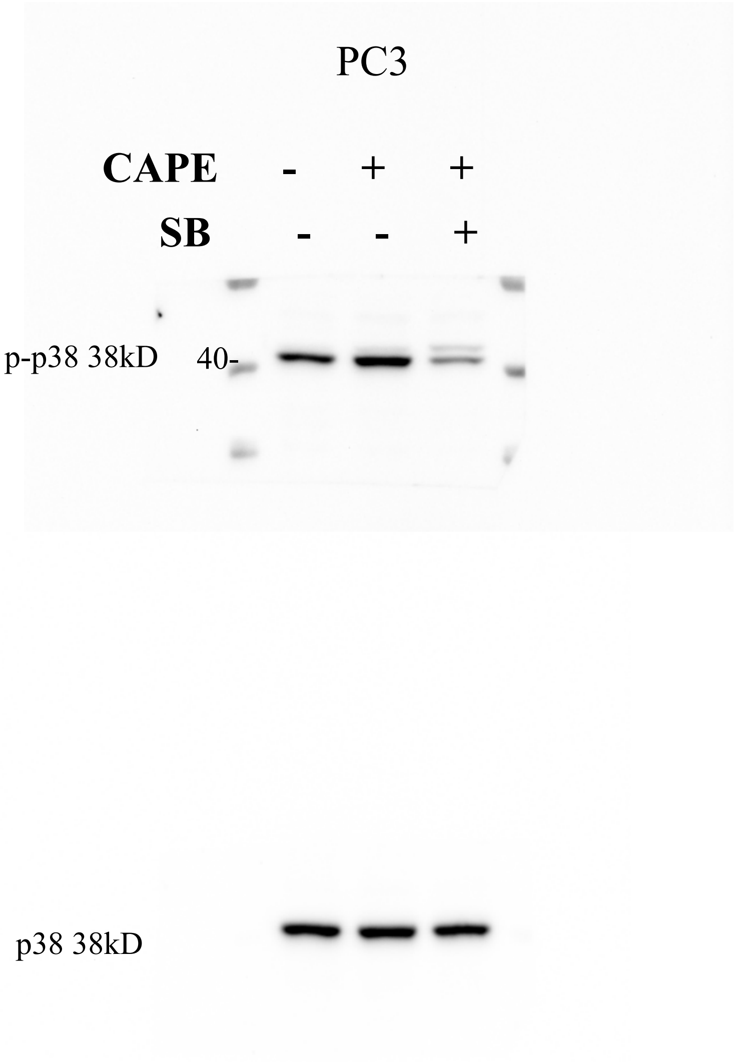
PC3
CAPE - + +
SB - - +
p-p38 38kD
40-
p38 38kD

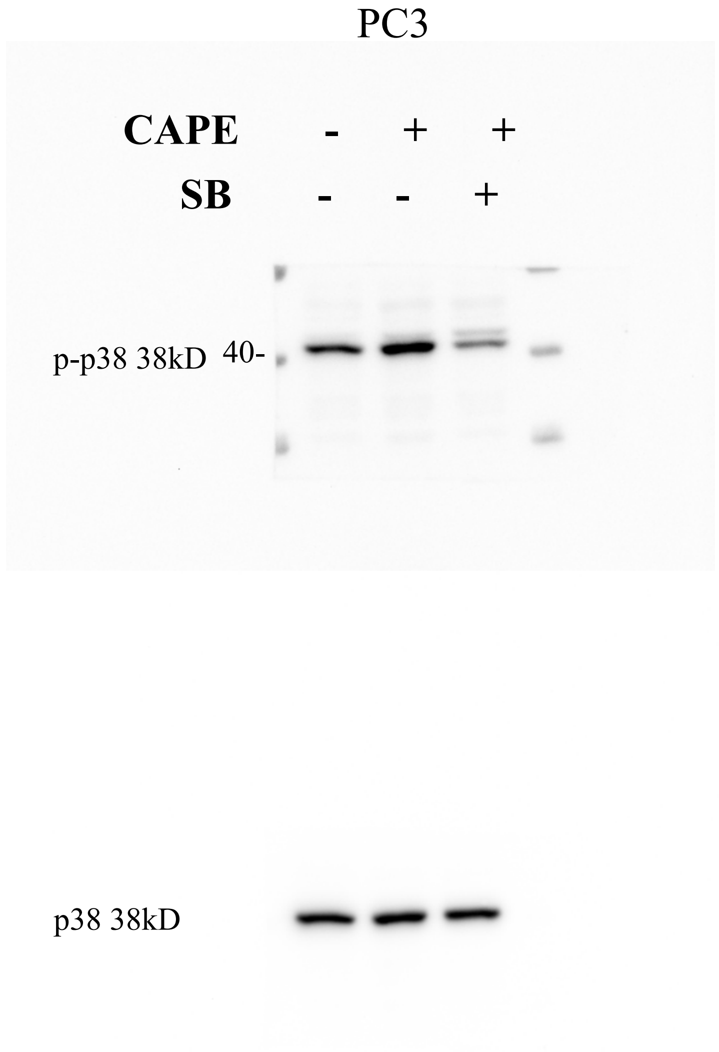
PC3
CAPE - + +
SB - - +
p-p38 38kD
40-
p38 38kD

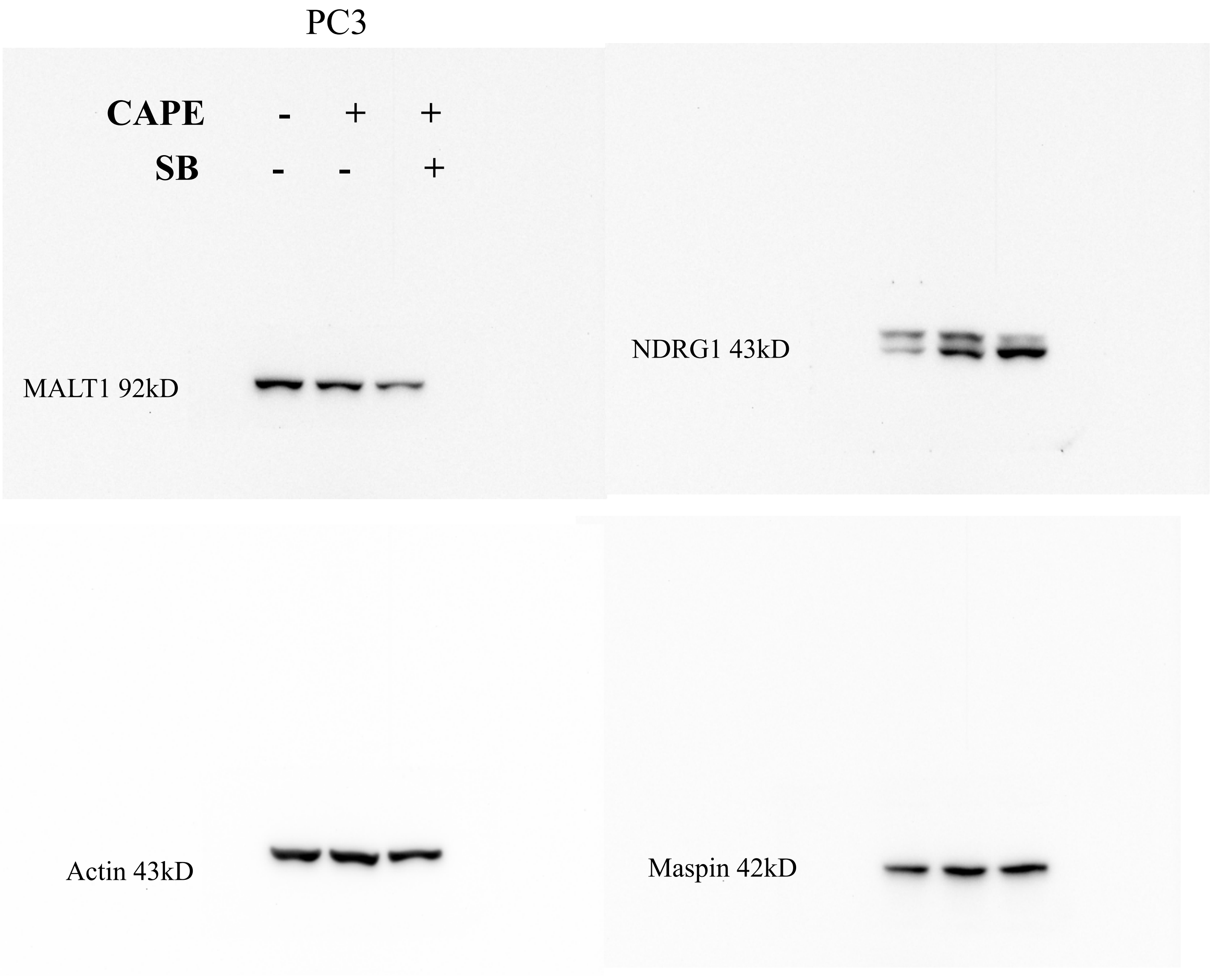
PC3
CAPE - + +
SB - - +
MALT1 92kD
NDRG1 43kD
Actin 43kD
Maspin 42kD

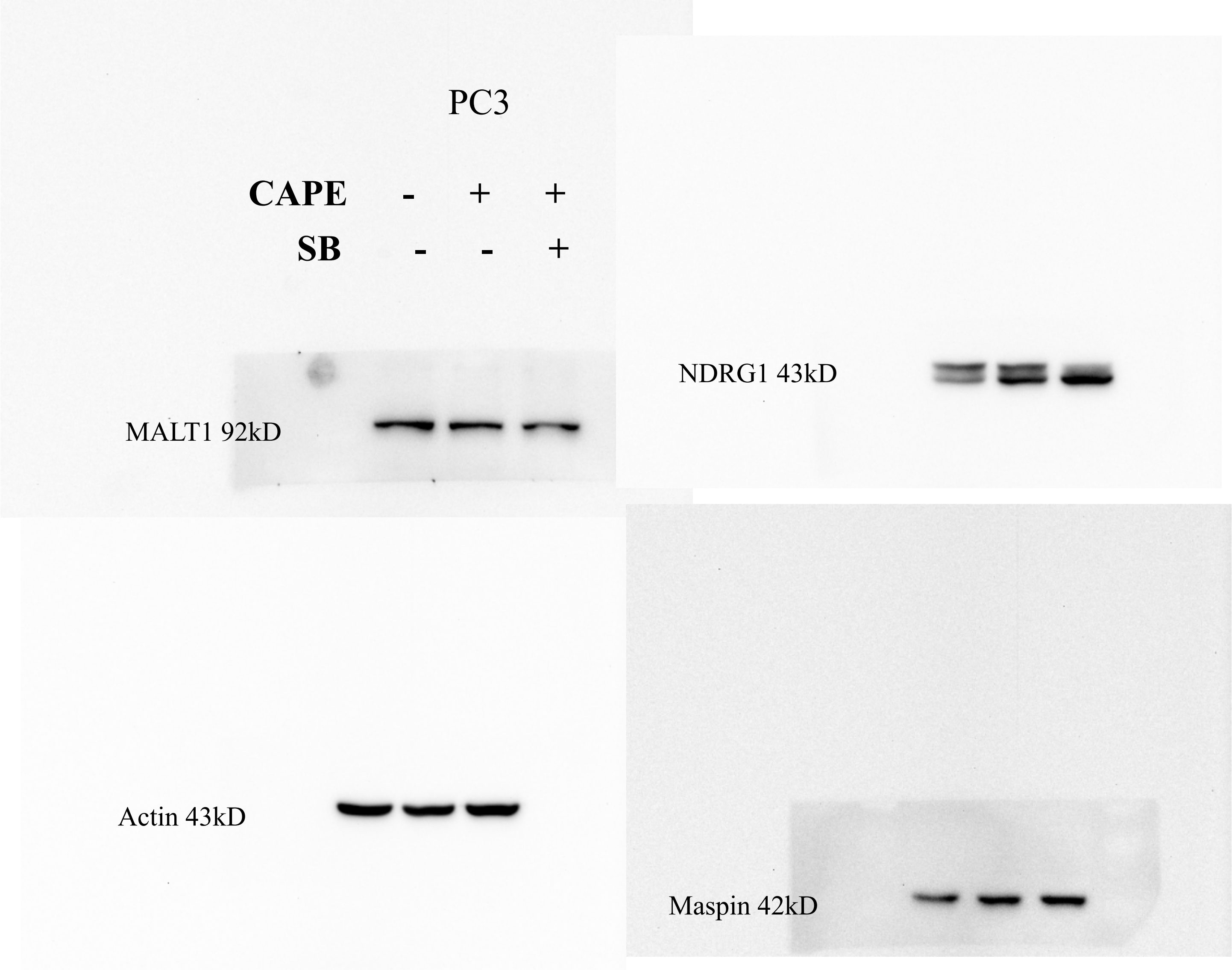
PC3
CAPE - + +
SB - - +
MALT1 92kD
NDRG1 43kD
Actin 43kD
Maspin 42kD

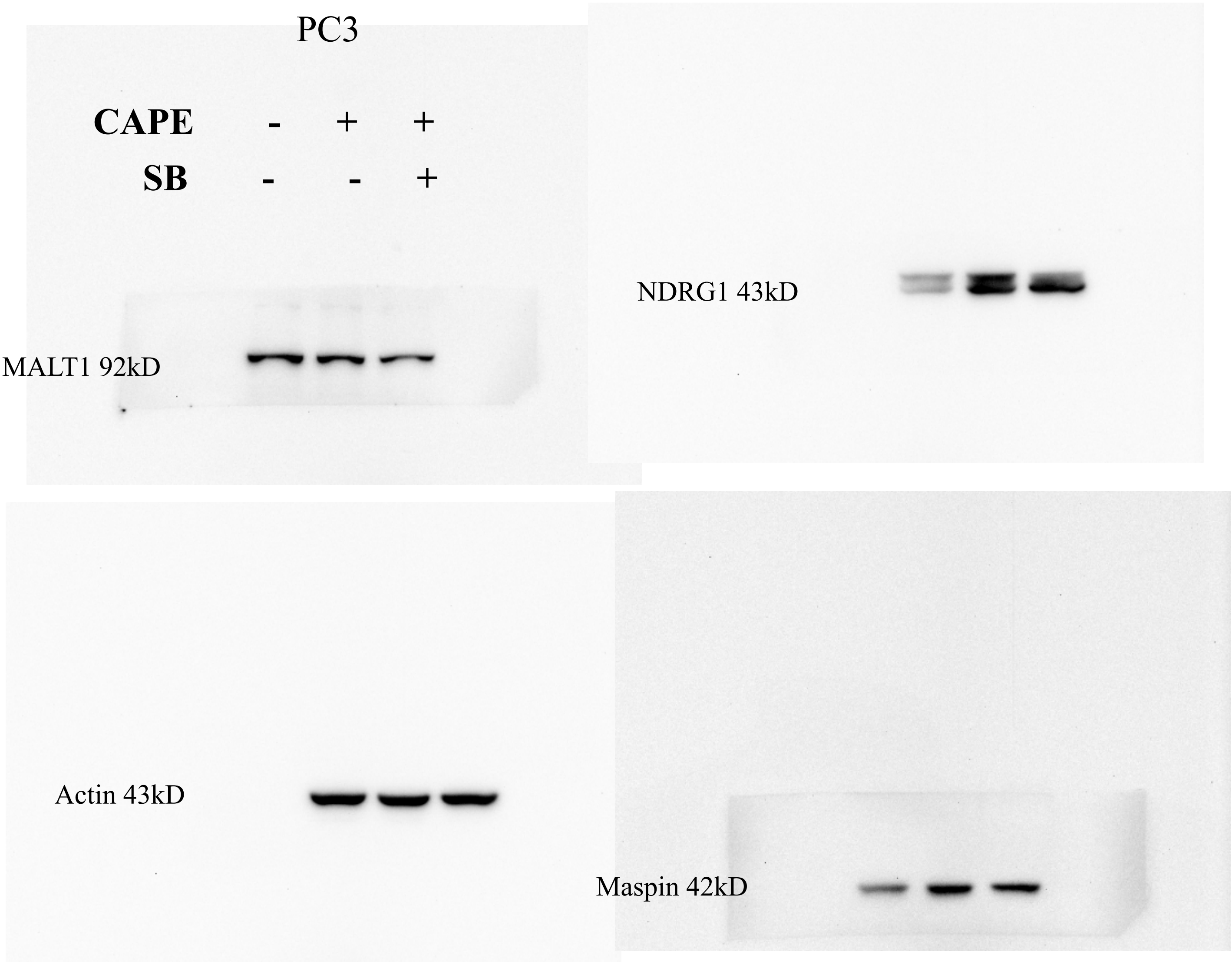
PC3
CAPE - + +
SB - - +
MALT1 92kD
NDRG1 43kD
Actin 43kD
Maspin 42kD

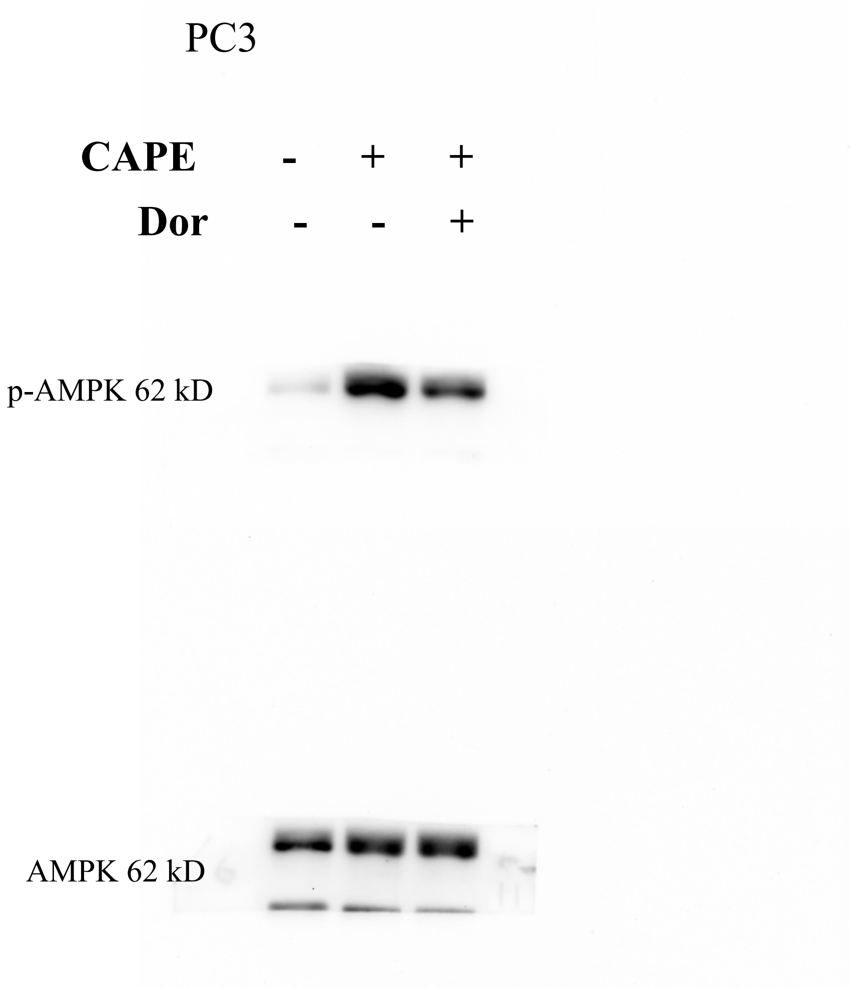
PC3
CAPE - + +
Dor - - +
p-AMPK 62 kD
AMPK 62 kD

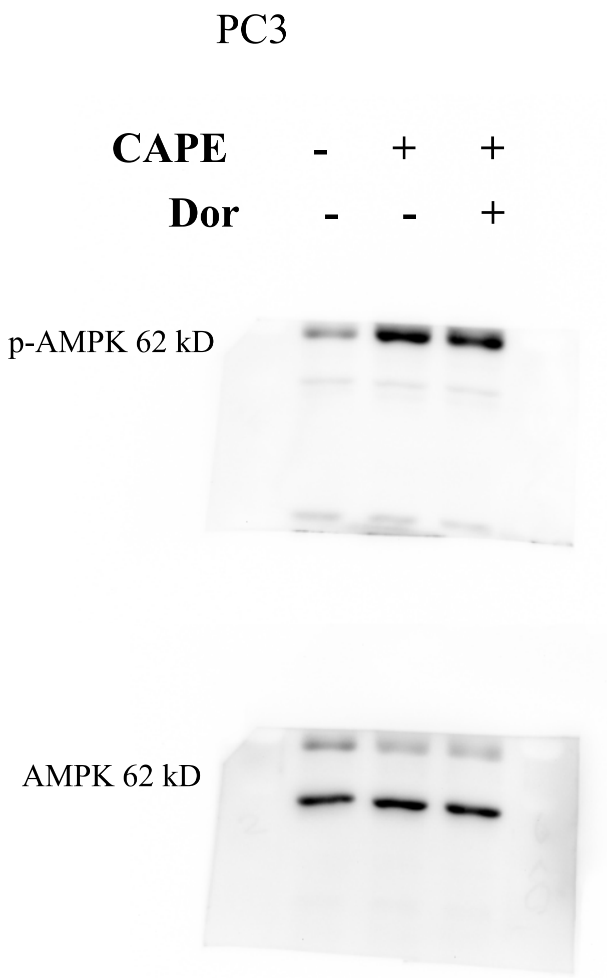
PC3
CAPE - + +
Dor - - +
p-AMPK 62 kD
AMPK 62 kD

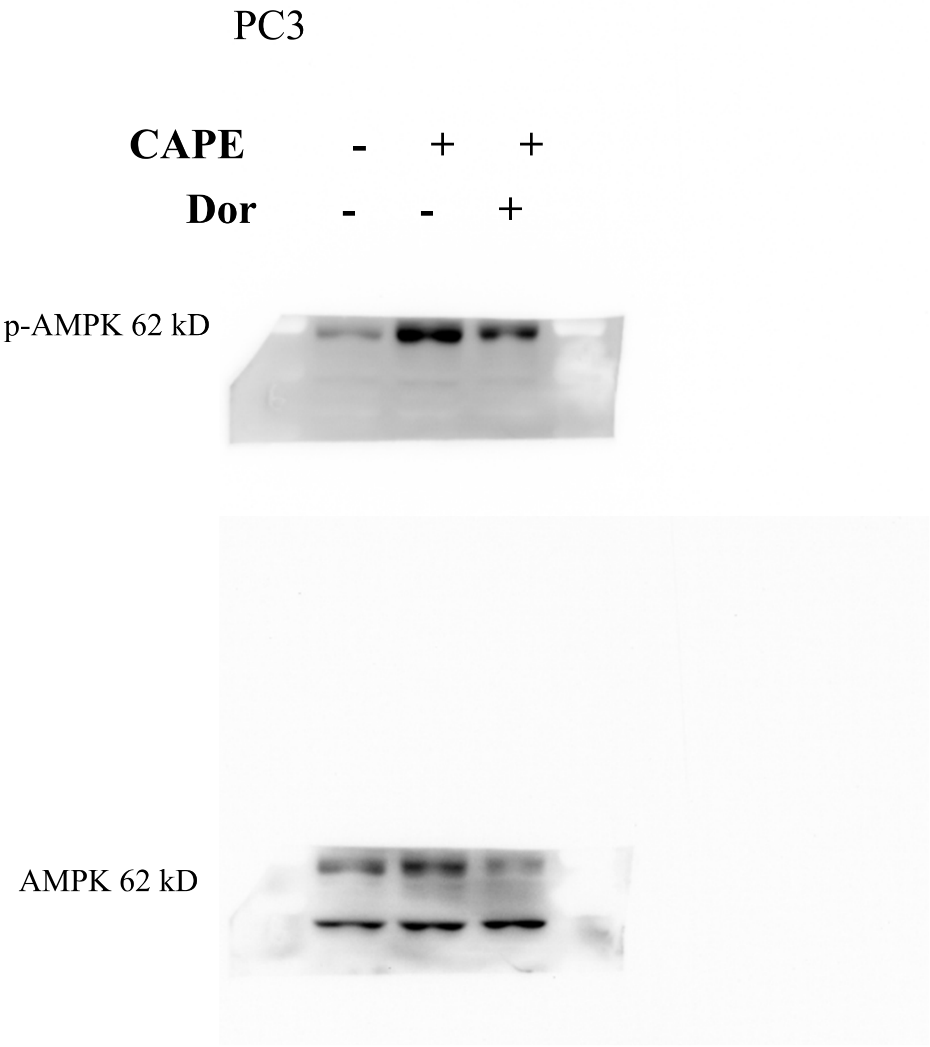
PC3
CAPE - + +
Dor - - +
p-AMPK 62 kD
AMPK 62 kD

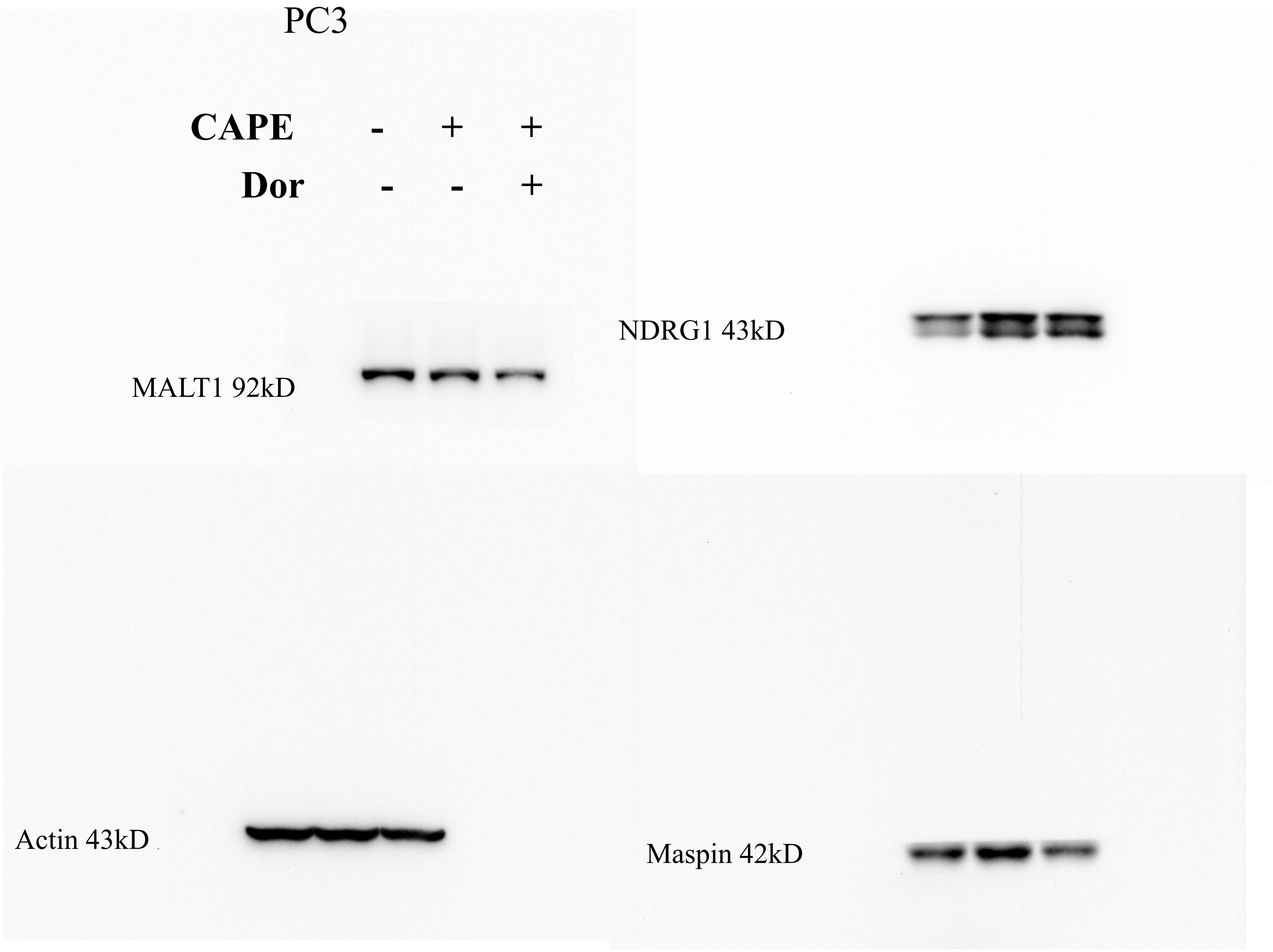
PC3
CAPE - + +
Dor - - +
MALT1 92kD
NDRG1 43kD
Actin 43kD
Maspin 42kD

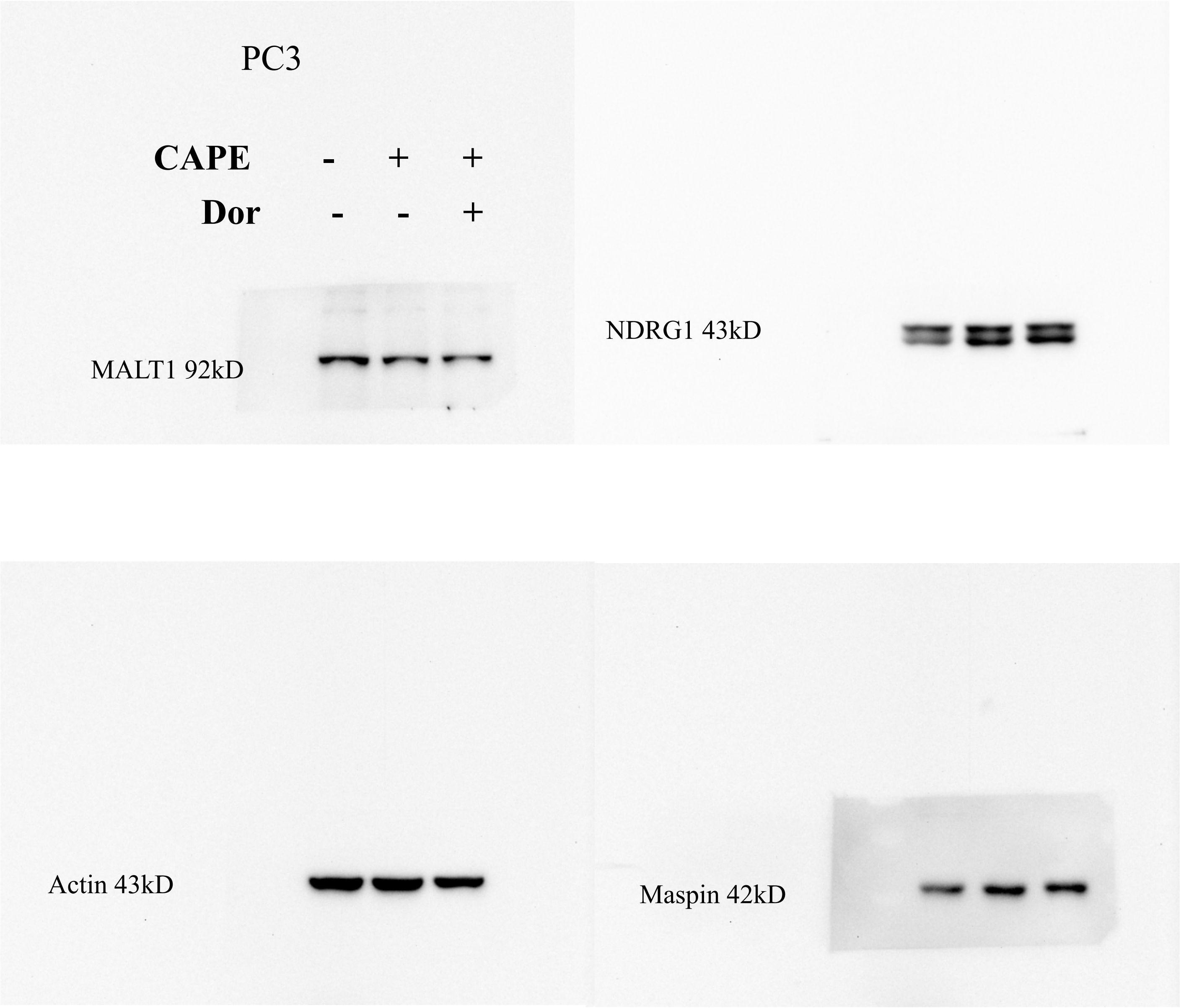
PC3
CAPE - + +
Dor - - +
MALT1 92kD
NDRG1 43kD
Actin 43kD
Maspin 42kD

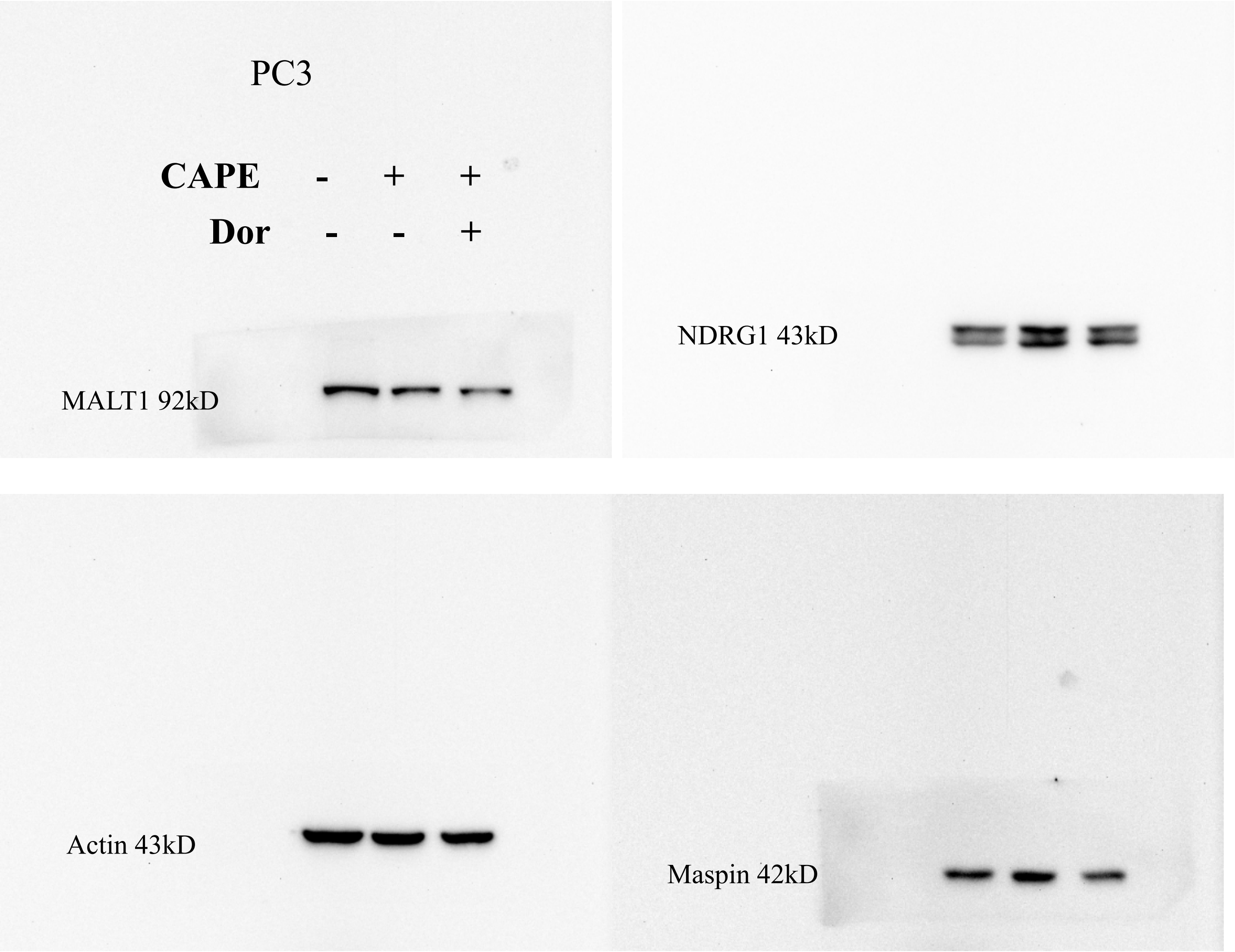


**Figure S5**: Original uncropped Western blots of figure 6
