# Supplementary figures and images for: The Antitumor Effect of Caffeic Acid Phenethyl Ester by Downregulating Mucosa-Associated Lymphoid Tissue 1 via AR/p53/NF-κB Signaling in Prostate Carcinoma Cells
Source: Cancers (Basel). 2022 Jan 6;14(2):274. doi: 10.3390/cancers14020274 (PMC8773797; doi:10.3390/cancers14020274)

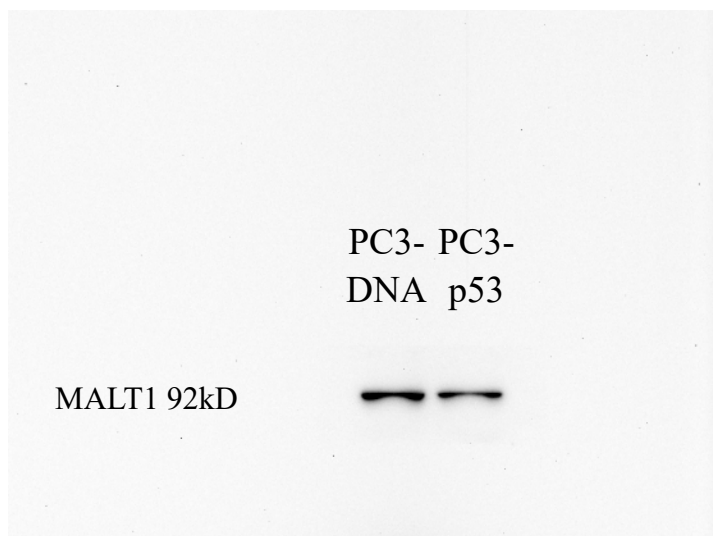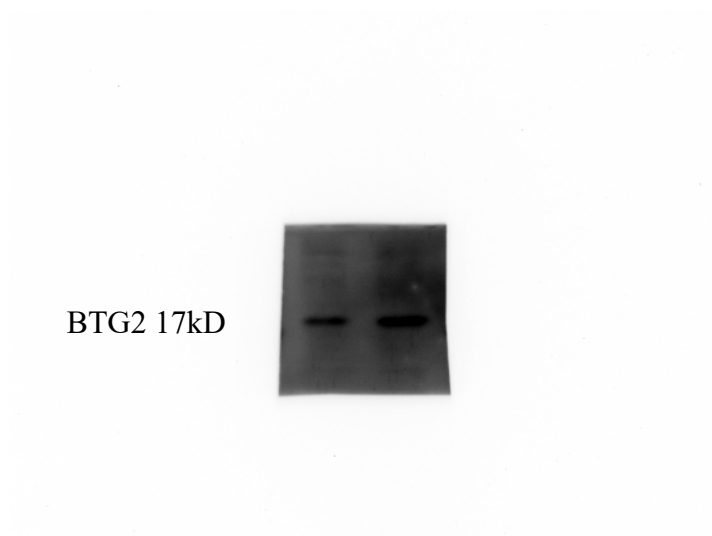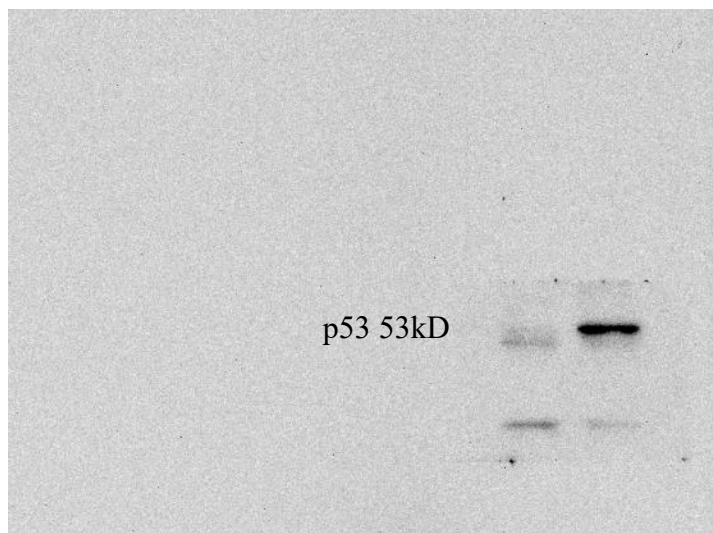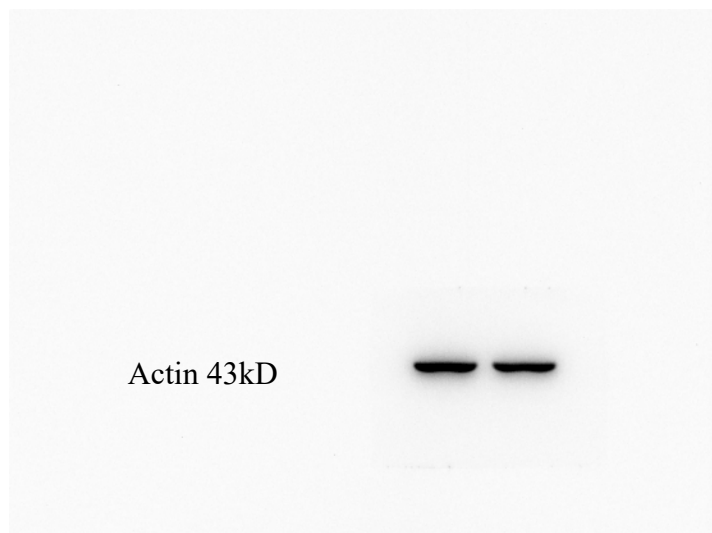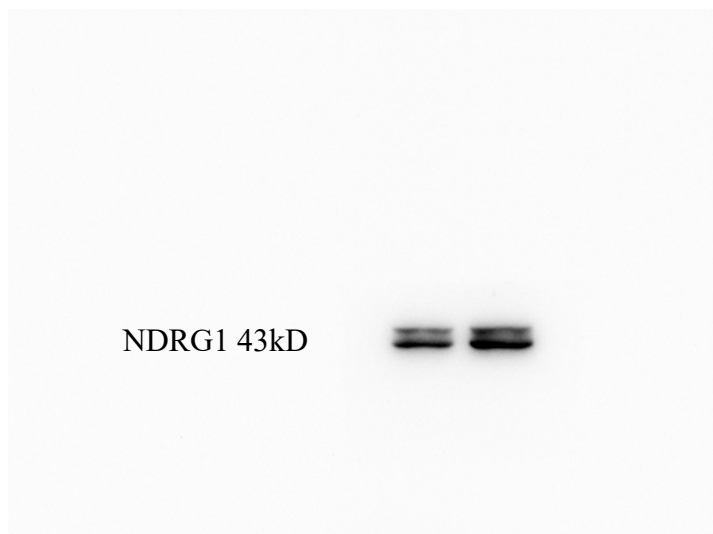

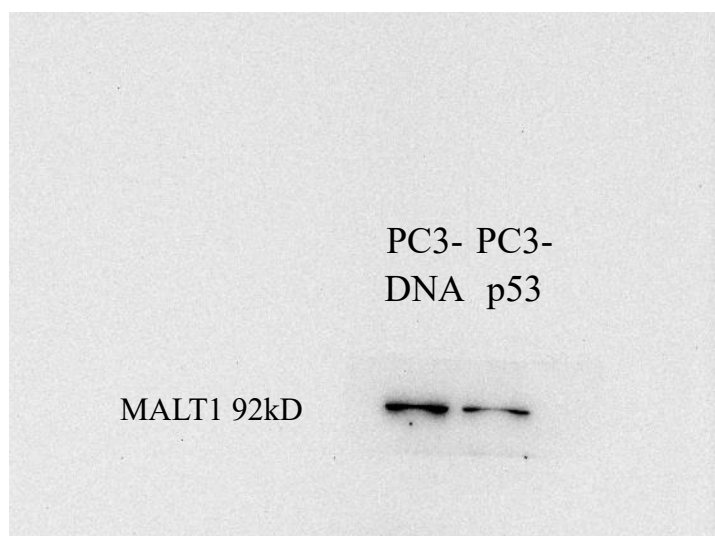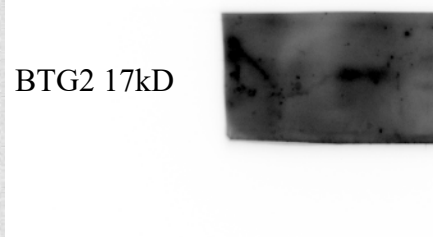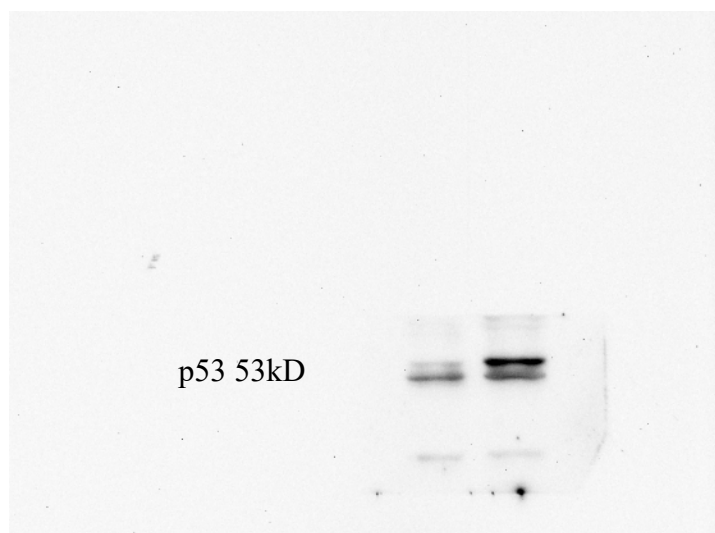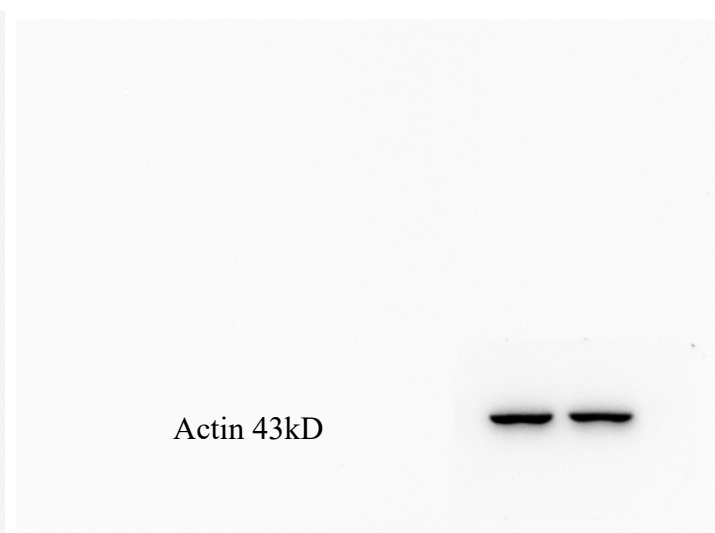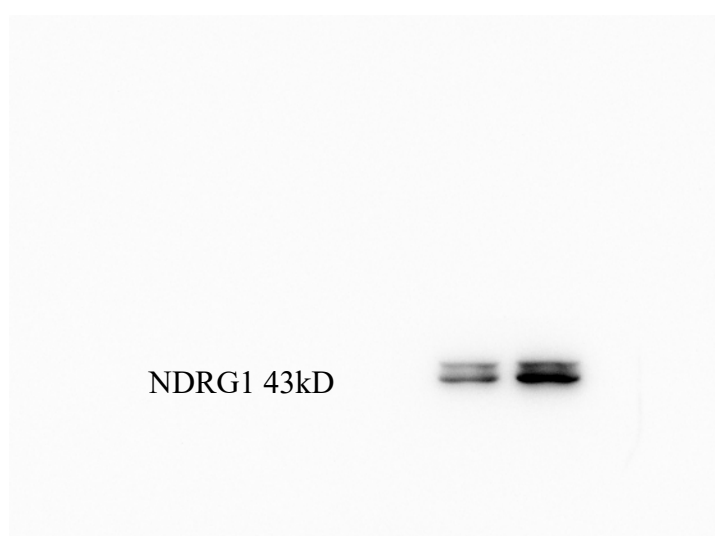

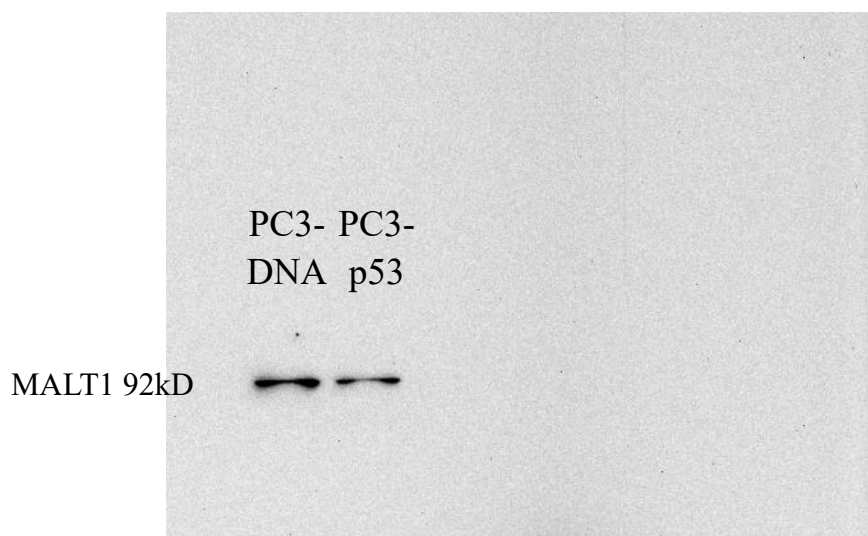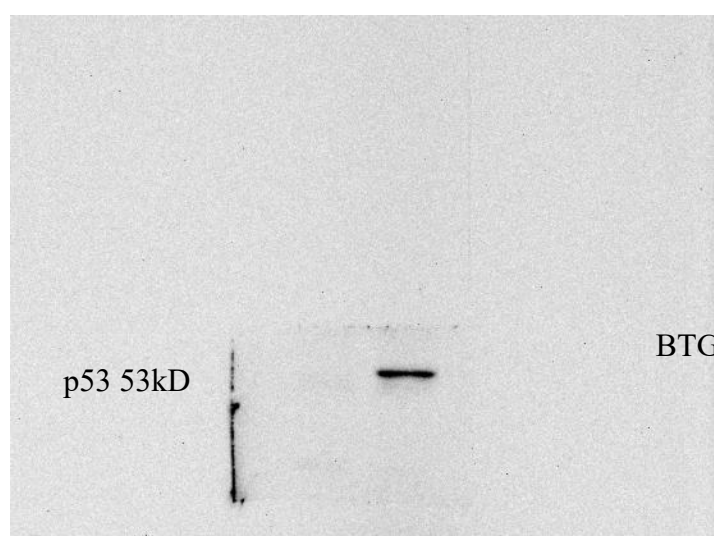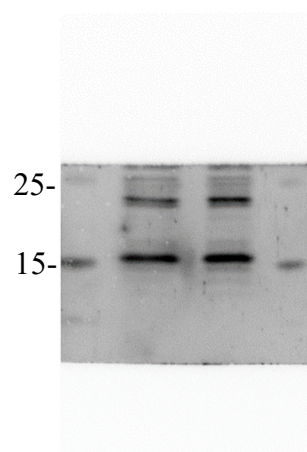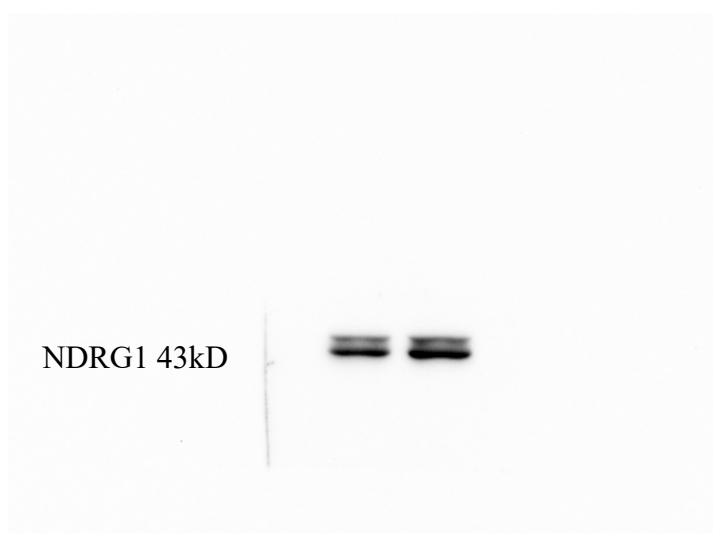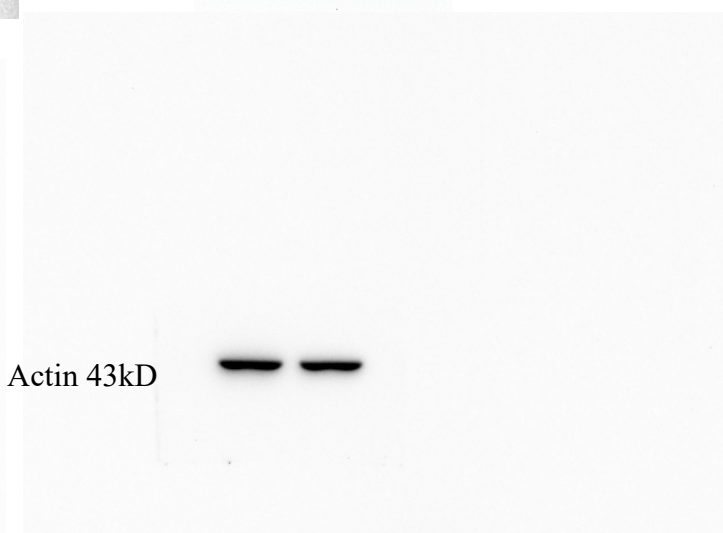

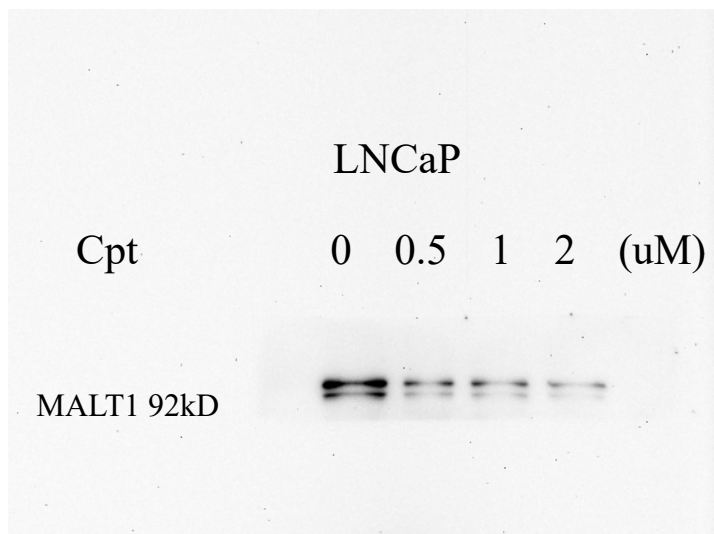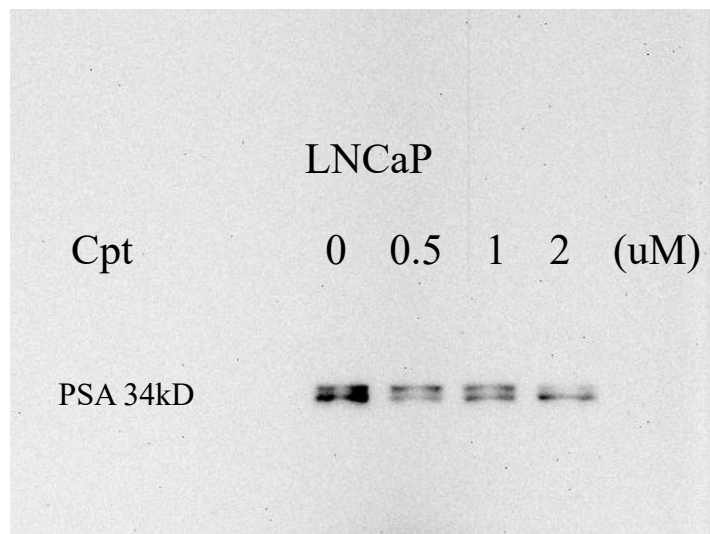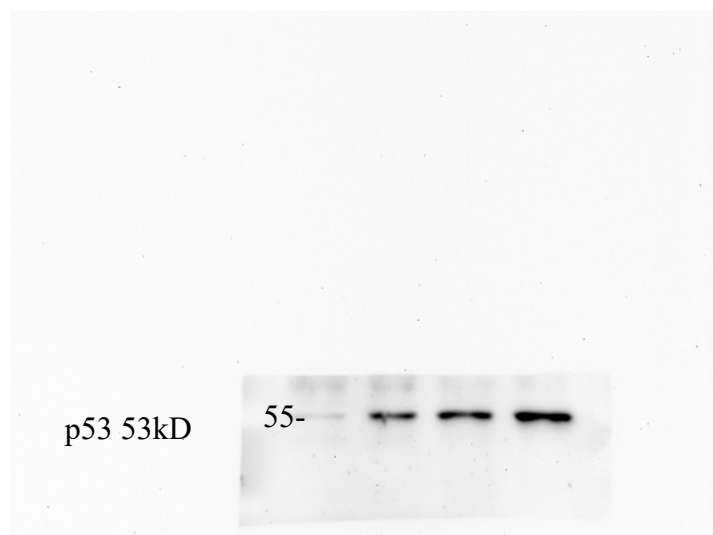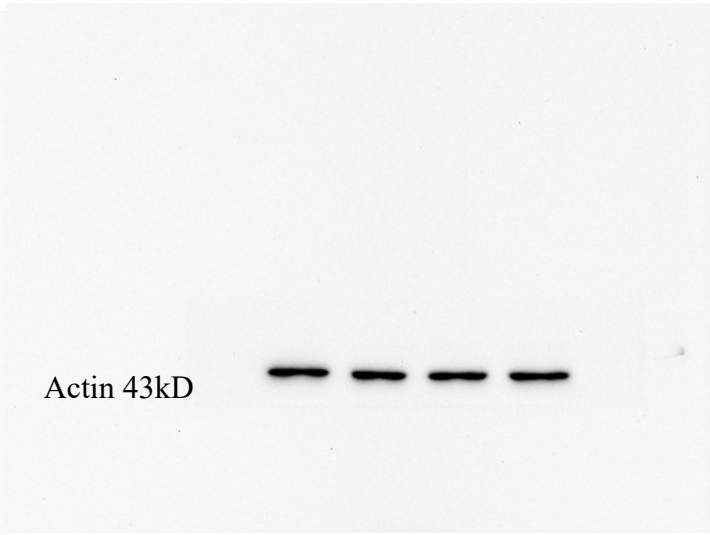

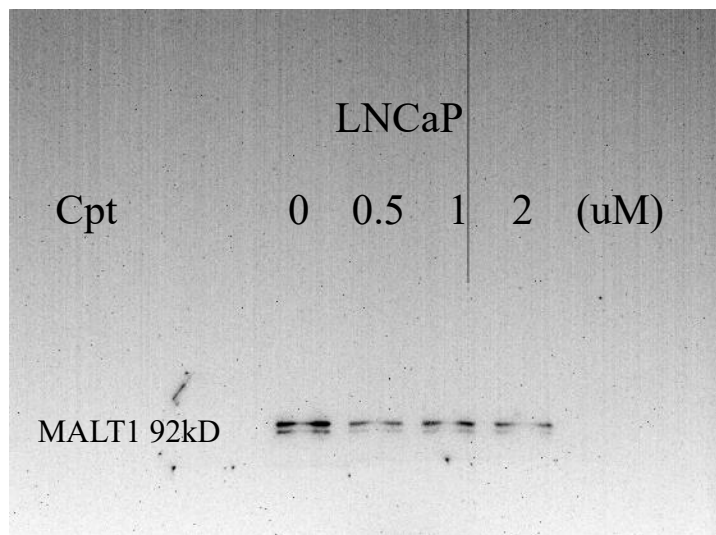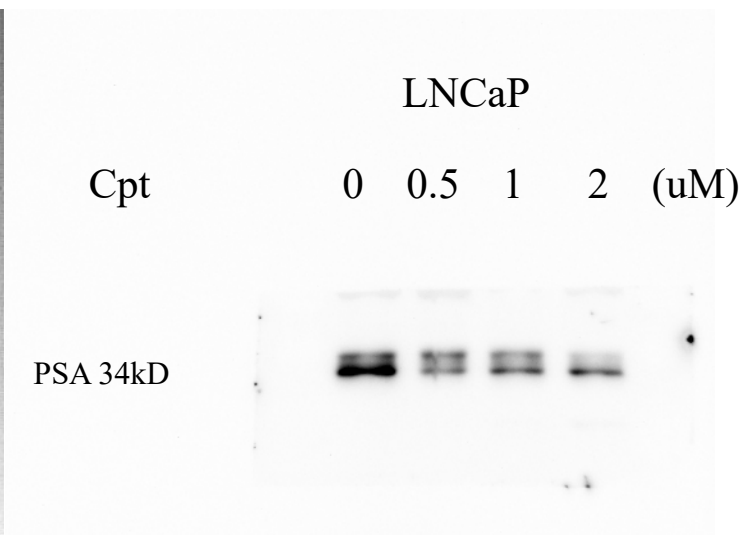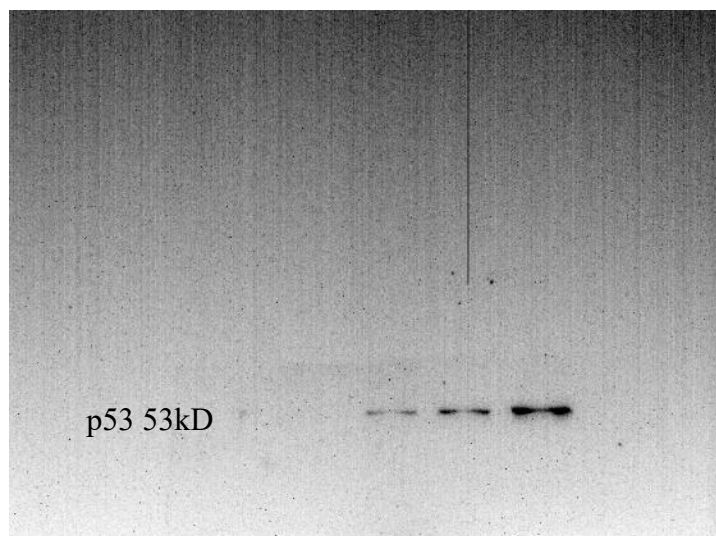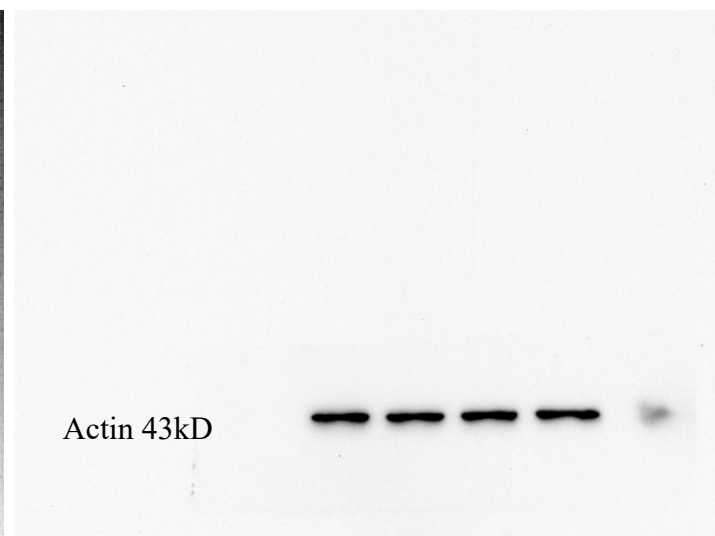

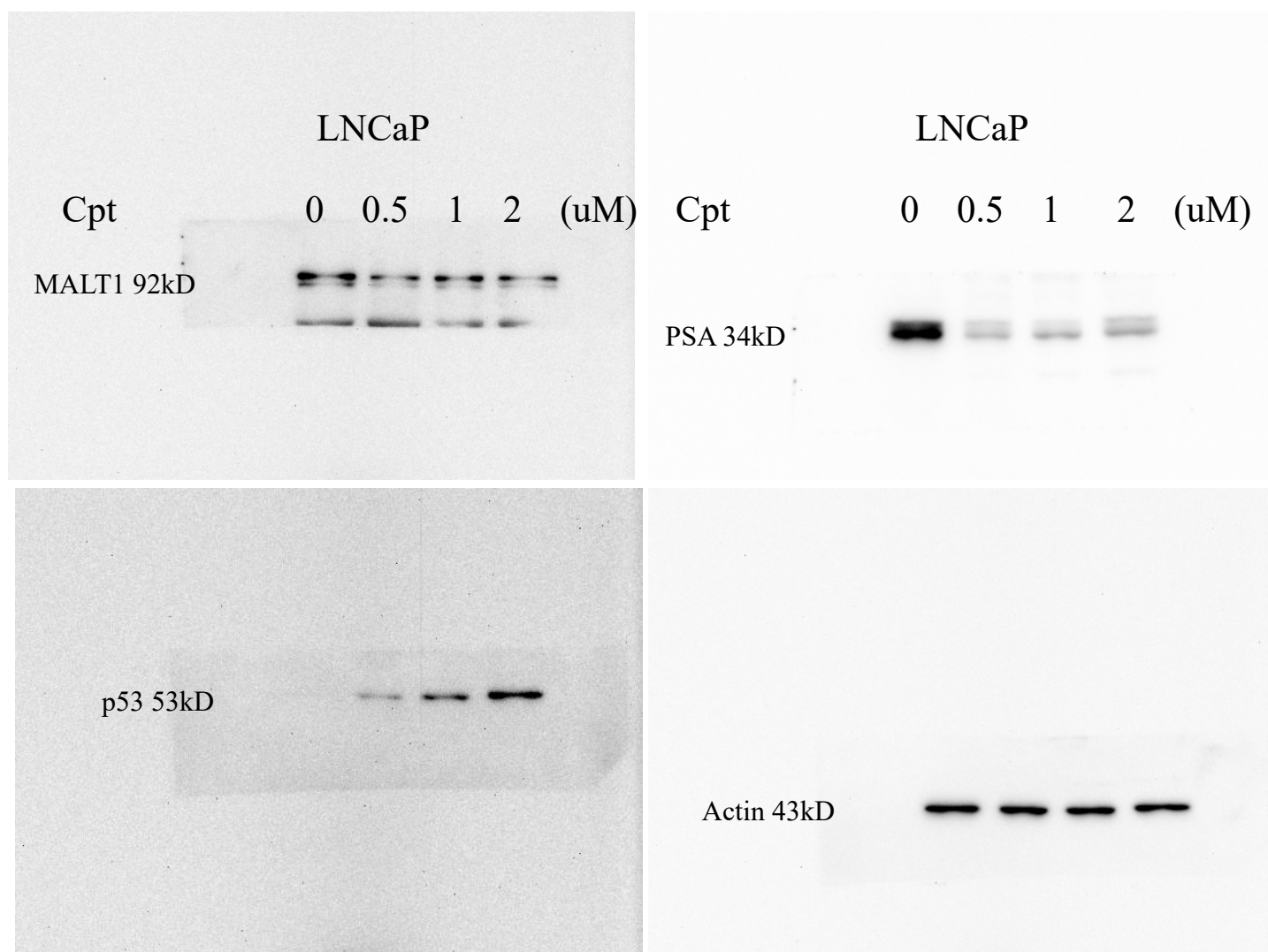

**Figure S2:** Original uncropped Western blots of figure 2

Supplement: Supplementary file 1 [file cancers-14-00274-s001.zip › cancers-1524042-supplementary/Figure S2.pdf]

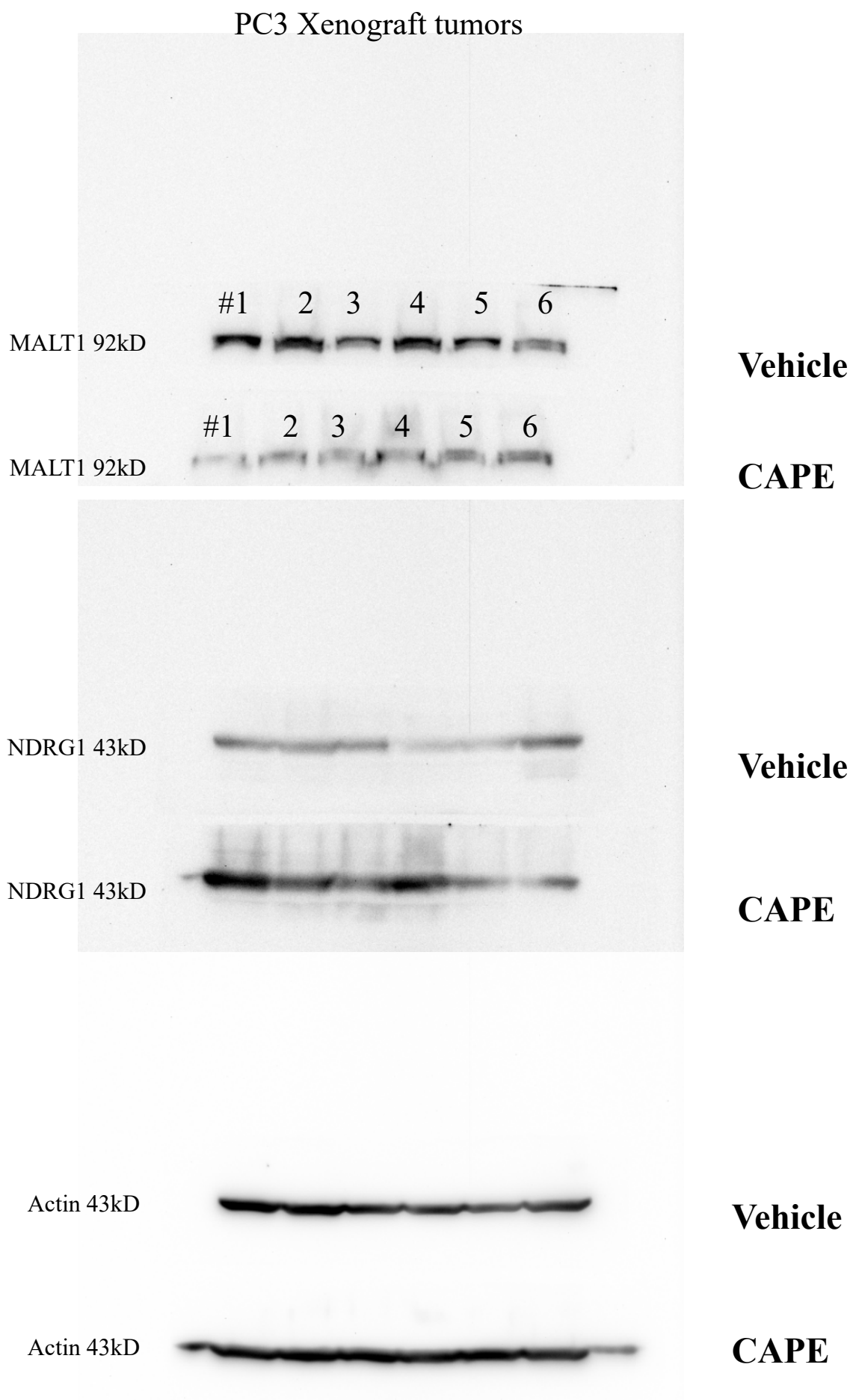

**Figure S6:** Original uncropped Western blots of figure 7

Supplement: Supplementary file 1 [file cancers-14-00274-s001.zip › cancers-1524042-supplementary/Figure S6.pdf]
